# New Brusatol Derivatives as Anti-Settlement Agents Against Barnacles, Targeting HSP90: Design, Synthesis, Biological Evaluation, and Molecular Docking Investigations

**DOI:** 10.3390/ijms26020593

**Published:** 2025-01-12

**Authors:** Wang Jiang, Tongtong Luan, Pei Cao, Zhonghui Ma, Zhiwei Su

**Affiliations:** 1 College of Agriculture, Guangxi University, Nanning 530004, China; jiangwang@st.gxu.edu.cn (W.J.); mazhonghui@gxu.edu.cn (Z.M.); 2 Traditional Chinese Herbal Medicine Resources and Agriculturalization Research Institute, Guangxi University, Nanning 530004, China; 3 Institute of Marine Drugs, Guangxi University of Chinese Medicine, Nanning 530200, China; luantongtong2021@stu.gxtcmu.edu.cn (T.L.); caopeicib@hotmail.com (P.C.)

**Keywords:** brusatol, derivatives, barnacle, antifouling, molecular docking

## Abstract

The increasing challenge of marine biofouling, mainly due to barnacle settlement, necessitates the development of effective antifoulants with minimal environmental toxicity. In this study, fifteen derivatives of brusatol were synthesized and characterized using ^13^C-NMR, ^1^H-NMR, and mass spectrometry. All the semi-synthesized compounds obtained using the Multi-Target-Directed Ligand (MTDL) strategy, when evaluated as anti-settlement agents against barnacles, showed promising activity. Compound **3** exhibited the highest anti-settlement capacity, with an EC_50_ value of 0.1475 μg/mL, an LC_50_/EC_50_ ratio of 42.2922 (>15 indicating low toxicity), and a resuscitation rate of 71.11%, while it showed no significant phenotypic differences in the zebrafish embryos after treatment for 48 h. The toxicity screening of zebrafish also demonstrated the low ecotoxicity of the selected compounds. Furthermore, homology modeling of the HSP90 structure was performed based on related protein sequences in barnacles. Subsequently, molecular docking studies were conducted on HSP90 using these newly synthesized derivatives. Molecular docking analyses showed that most activated derivatives displayed low binding energies with HSP90, aligning well with the biological results. They were found to interact with key residues in the binding site, specifically ARG243, TYR101, and LEU73. These computational findings are anticipated to aid in predicting the enzyme targets of the tested inhibitors and their potential interactions, thus facilitating the design of novel antifoulants in future research endeavors.

## 1. Introduction

Marine biofouling occurs when marine invertebrates, such as barnacles, bryozoans, and tubeworms, attach to artificial structures like ships and offshore platforms [1]. This attachment leads to physical damage and increased maintenance costs, rising energy consumption, and accelerated corrosion, resulting in global economic losses of over one hundred billion dollars each year [2,3]. Since the mid-20th century, antifouling efforts have focused on toxic coatings that release metal ions and biocides to deter or eliminate these organisms [4]. However, these methods harm non-target marine life and can contaminate the food chain due to bioaccumulation [5]. As a result, recent research has shifted toward developing eco-friendly antifoulants [6,7,8,9]. Most natural products derived from marine sources have been found to exhibit antifouling properties [9,10]. This occurs because they enhance the lipophilicity of molecules or disrupt biological attachment, particularly through specific functional groups, including pyridine, halogens, esters, and furanone rings [9,10,11]. Notably, some compounds from terrestrial plants also exhibit strong antifouling potential, including capsaicin, cypermethrin, juvenoids, terpenes, alkaloids, flavonoids, tannins, and others [10,11,12,13,14]. Terrestrial plants can be sustainably propagated to produce high-yield compounds, making them promising candidates for environmentally friendly antifoulants. Uncovering more potent antifouling molecules with well-defined mechanisms is crucial for the advancement of new antifoulants in the future.

The Multi-Target-Directed Ligand (MTDL) strategy, which combines different bioactive ligands or pharmacophores into a single molecule, has been demonstrated to improve drug leads, for example, in the treatment of neurodegenerative disorders [15]. Takamura et al. first applied the MTDL strategy in marine antifouling, creating hybrid compounds of butenolide and geraniol that inhibit *Balanus amphitrite* cyprid larvae settlement at lower concentrations than the individual components [16]. The larvae of many sessile marine invertebrates undergo complex processes of settlement and metamorphosis, mediated by various signaling pathways [17,18]. Heat shock protein 90 (HSP90) in marine invertebrates is a crucial and highly conserved protein that plays a significant role in the processes of metamorphosis and settlement. Its importance in these developmental stages is well established [17,18,19]. Qian et al. found that barnacle larvae exposed to antifoulants containing butanolide had higher levels of HSP90, which could disrupt the larvae’s ability to grow and settle properly [17]. In 2023, Adela Jing Li et al. found that exposure to 100 µg Cu/L at 20 °C significantly upregulated HSP90 genes in the marine copepod *Tigriopus japonicus* [19]. The NO/cGMP signaling pathway has been shown to play a critical role in the morphological development of invertebrates, affecting their settlement [20]. Specifically, HSP90 acts as a main molecular chaperone, facilitating the downstream NO synthase that is activated or expressed [21,22,23,24]. This results in the enhanced biosynthesis of cyclic guanosine phosphate (cGMP), which ultimately activates downstream regulators, thereby initiating related programs that inhibit larval attachment and deformation [20,21,22,23,24].

Brusatol, a well-researched natural compound from *Brucea javanica*, is known for its various pharmacological effects, including anti-cancer, anti-HIV, anti-malaria, anti-neoplasm, and so on [25]. Research has shown that brusatol and bruceantin target HSP90 to exert activity against pancreatic cancer and castration-resistant prostate cancer, respectively [26,27]. Intriguingly, in our initial research, we unexpectedly discovered that terrestrial-derived quassinoids effectively prevent barnacle larvae from settling [28]. Additionally, we conducted homology modeling to generate the three-dimensional structure of the HSP90 homologous sequence from barnacles (*Balanus amphitrite*), which exhibits high homology to human HSP90 (PDB: 8ffw) with a sequence identity of 81.99% [29]. Based on this discovery, our team has initiated a series of studies to further investigate the potential of brusatol in marine antifouling. The primary task is to use computational approaches and the MTDL strategy to create brusatol hybrid molecules at the C-15 position by adding functional groups from marine sources, including flexible side chains, phenyl-substituted side chains, and heterocyclic groups, to develop more brusatol derivatives that work better and are less toxic. Molecular docking studies are then performed using the HSP90 model, yielding preliminary insights into the potential mechanism of action of these compounds on the protein. As part of our continuing efforts to develop 15 brusatol hybrid derivatives as new potent antifoulants against barnacles with low binding energies with HSP90, this study unveils a new use for brusatol derivatives in combating biofouling, providing new perspectives in the exploration and development of traditional terrestrial natural products.

## 2. Results and Discussion

**Figure 1:**
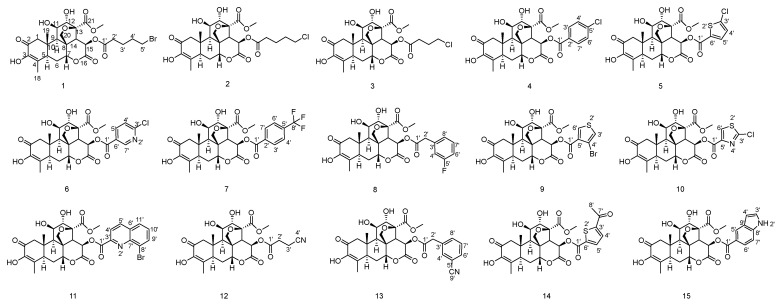
Structures of newly modified brusatol derivatives (compounds **1**–**15**). The numbers (e.g., 1, 2, 1′, 2′) in the figure represent the atomic numbering of the compound, which corresponds to the assignments in the NMR data.

**Scheme 1:**
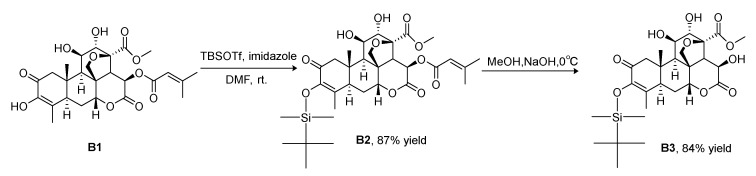
Semi-synthesis of compound **B3**. **B1** represents brusatol, while **B2** and **B3** are key intermediates involved in the structural modification process.

The quassinoid analogs **1**–**15** (Figure 1) were synthesized from the natural compounds of brusatol (**B1**) (Scheme 1), which were extracted from the dried fruits of *Brucea javanica*.

### 2.1. Chemical Synthesis

The modification pathways for the obtaining of brusatol derivatives **1**–**15** are outlined in Scheme 1, Scheme 2, Scheme 3 and Scheme 4. The experimental details and reaction yields are provided in Section 3. The ^1^H NMR, ^13^C NMR, and mass spectrometry data for the starting material brusatol (**B1**), as well as for the intermediate compounds **B2** and **B3**, were consistent with previously reported values in the literature [30,31]. Additionally, to supplement the available spectroscopic data for the derivatives, ^1^H and ^13^C NMR experiments were conducted, as shown in Figures S1–S36.

**Scheme 2:**
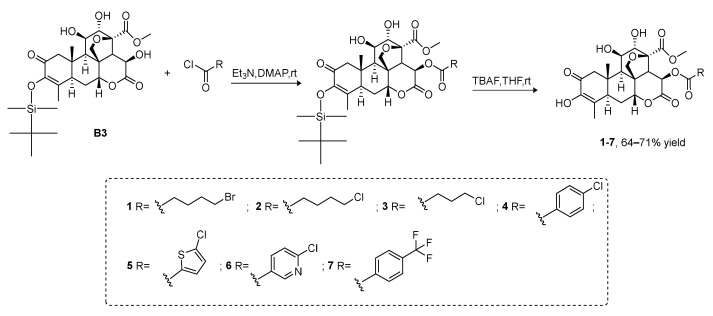
Semi-synthesis of compounds **1**–**7**.

**Scheme 3:**
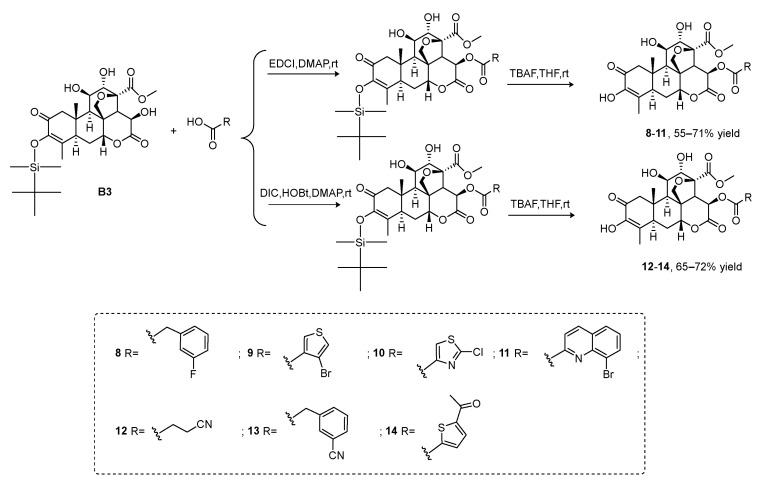
Semisynthesis of compounds **8**–**14**.

**Scheme 4:**
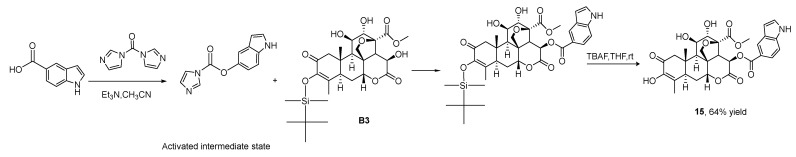
Semisynthesis of compound **15**.

In this study, we modified 15 brusatol derivatives at the C-15 position via a four-step synthetic sequence. The 3-OH group was initially protected using chlorosilane TBMSOTF following reported procedures in Scheme 1, yielding intermediate compound **B2**. We also optimized the existing protocol [30] to eliminate the final purification step and improve the overall yields of the target compounds. By taking advantage of the significantly reduced water solubility after silicon protection, we developed an efficient and cost-effective purification method using ethanol and water recrystallization. This approach greatly enhanced production efficiency and made the reaction process more environmentally friendly. Secondly, this was followed by hydrolytic cleavage of the side chain at C-15, yielding a 15-OH intermediate (**B3**) (Scheme 1). Thirdly, we employed various amide synthesis techniques to structurally modify the C-15 position to produce the desired derivatives (Scheme 2, Scheme 3 and Scheme 4). The final step entailed the deprotection of 3-OH through desilylation, successfully generating target compounds **1**–**15** (Scheme 2, Scheme 3 and Scheme 4). Moreover, the third step incorporated an array of amide bond formation strategies to refine the synthesis pathway. We explored a range of amide synthesis experiments to identify the optimal reaction conditions for the formation of brusatol derivatives [32,33,34,35,36,37,38,39,40,41,42,43]. We discovered that substrate suitability varied across different amide coupling protocols, as did the required quantity of catalyst equivalents when dealing with diverse small-molecule substrates. This variability can likely be attributed to the substrates’ chemical characteristics. For instance, a common trend observed in all successful reactions in this paper is that molecules with higher electronegativity, which exhibit electron-withdrawing properties, tended to react more readily. Conversely, electron-donating substrates necessitated a substantial increment in catalyst equivalents or a prolonged reaction duration to achieve the desired outcomes. Indeed, several less electronegative small molecules did not partake in the reaction at all. We conducted numerous experiments and found that many small molecules with weaker electronegativity, such as Oxazole-4-carboxylic acid, pyrimidine-5-carboxylic acid, quinoline-5-carboxylic acid, 4-morpholinecarbonyl chloride, 1-pyrrolidinecarbonyl chloride, thiazole-4-acetic acid, 2-mercapto-4-methyl-5-thiazoleacetic acid, 3-acetylbenzoic acid, cinnamoyl chloride, 1-piperidinepropanol, 4-benzylpiperidine, and 2-phenylquinoline-4-carboxylic acid, failed to react under various catalytic systems. This prevented the formation of the desired products. Electron-withdrawing effects or electronegativity have been shown to play a pivotal role in amide reactions. Specifically, studies suggest that electronegative or electron-withdrawing groups enhance the electrophilicity of the carbonyl carbon while diminishing the stabilizing influence of the nitrogen lone pair, thus facilitating amide activation [44]. Similarly, Wang et al. demonstrated that these electron-withdrawing effects increase the protonation capacity of reactants, which in turn accelerates reaction rates and effectively promotes esterification [45]. Our experimental results align with these observations, revealing that carboxylic acid molecules with higher electronegativity tend to react more readily with the 15-OH of brusatol. This finding provides additional experimental evidence that underscores the critical role of electronic effects in both amide activation and esterification processes.

### 2.2. Screening of Anti-Larval Settlement Effects of Brusatol Derivatives Against Balanus Amphitrite

**Figure 2:**
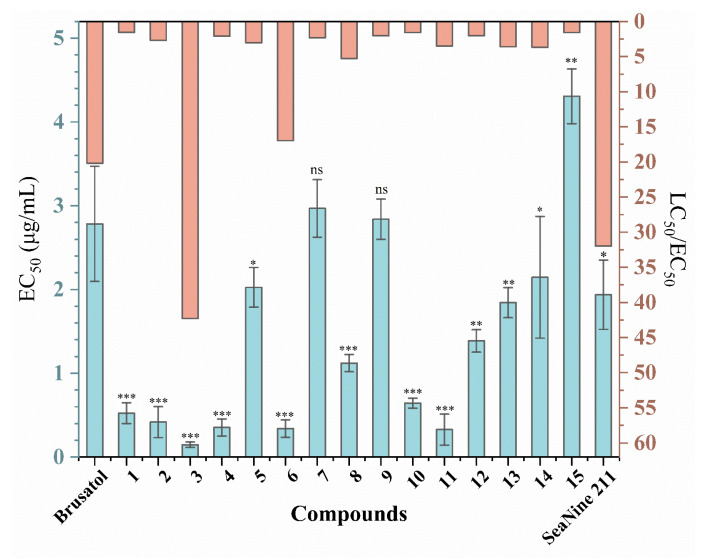
Anti-larval settlement effects of brusatol derivatives against *Balanus amphitrite*. SeaNine 211 is used as a positive control. LC_50_/EC_50_ values represent the toxicity level, with a ratio greater than 15 indicating lower toxicity. Statistical significance compared to brusatol is indicated as follows: *p* < 0.05 (*), *p* < 0.01 (**), *p* < 0.001 (***), and *p* > 0.05 (ns).

In this study, derivative **3** exhibited superior activity and lower toxicity, with an EC_50_ value of 0.1475 μg/mL, compared to the original compound brusatol (2.7826 μg/mL) and the positive control SeaNine 211 (1.9363 μg/mL). The LC_50_/EC_50_ ratio for this compound was 42.2922, reflecting its very low toxicity. Similarly, derivative **6** displayed an EC_50_ value of 0.3402 μg/mL and an LC_50_/EC_50_ ratio of 16.9633 (with >15 indicating low toxicity) [9], also demonstrating high activity and reduced toxicity. The efficacy of structural modification through the incorporation of chlorine side chains significantly enhances the bioactivity of these compounds while effectively mitigating toxicity, as demonstrated by the success of these two compounds. In contrast, while derivatives **4** and **11** demonstrated relatively high activity (EC_50_ values of 0.3527 and 0.3274 μg/mL), their LC_50_/EC_50_ ratios of 2.1018 and 3.4814 suggest increased toxicity, thereby limiting their potential for practical application. Additionally, other derivatives exhibited considerable activity and displayed effective anti-settlement properties, though with varying levels of toxicity (Figure 2 and Table 1).

In summary, the exceptional performance of derivatives **3** and **6** confirmed the effectiveness of the structural modification design, offering valuable insights for the future development of low-toxicity and high-efficiency antifoulants.

### 2.3. Resuscitation Experiments on Brusatol Derivatives

**Figure 3:**
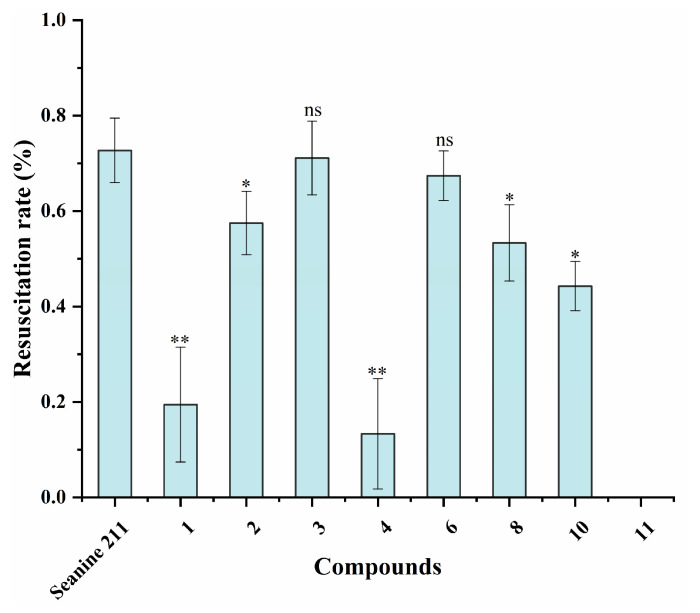
Rescue assays of *Balanus amphitrite* cyprid larvae, treated with compounds (1 μg/mL) for 24 h, rinsed three times with filtered seawater, and transferred to a 24-well plate containing 1 mL filtered seawater, with results observed after 48 h. Statistical significance compared to SeaNine 211 is indicated as follows: *p* < 0.05 (*), *p* < 0.01 (**), and *p* > 0.05 (ns).

This study evaluated the discovery of settlement ability in cyprid larvae that survived but remained unattached after drug treatment. Resuscitation experiments were conducted using eight brusatol derivatives (**1**–**4**, **6**, **8**, **10**, **11**) at a concentration of 1 μg/mL which showed significantly higher activity compared to brusatol (**B1**) (*p* < 0.001) (Figure 2). The proportion of larvae that regained settlement ability was defined in terms of the recovery rate [46]. The results were consistent with toxicity predictions based on LC_50_/EC_50_ values obtained from anti-settlement activity assays, enhancing the reliability of the findings. As shown in Figure 3, compounds **3** and **6** exhibited high recovery rates, suggesting that their effects on cyprid larvae may be more anesthetic in nature rather than toxic. This implies that these compounds have relatively low toxicity and could be safer for non-target organisms. Therefore, they present potential for further development and utilization. In contrast, compound **11** demonstrated a recovery rate of zero at 1 μg/mL, indicating a stronger toxic effect, which could limit its broader application due to the higher risk of harm to marine ecosystems.

### 2.4. Toxicity Screening of Derivatives Using Zebrafish Embryos

Compounds **3** and **6**, as the most active derivatives with low toxicity toward the target organism, underwent further toxicity screening using zebrafish embryos. The LC_50_ values at 48 h post-fertilization of compounds **3** and **6** were determined to be 15.7692 μg/mL and 8.1846 μg/mL, respectively, both of which are higher than that of the traditional antifouling agent SeaNine 211 (7.2236 μg/mL), indicating lower toxicity. Moreover, no significant phenotypic differences were observed between the treated zebrafish embryos and the control group at 48 h post-fertilization.

### 2.5. Homologous Modeling Analysis of HSP90

**Figure 4:**
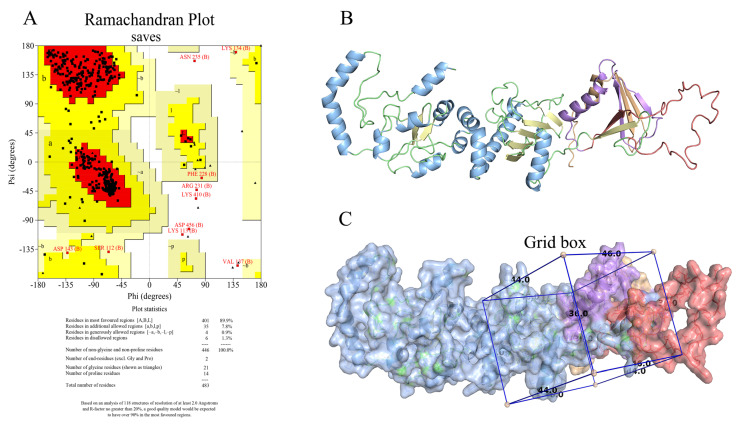
Homology modeling and structural analysis of HSP90. (**A**): Ramachandran plot analysis of 3D structural evaluation for the protein HSP90; (**B**): cartoon representation of the HSP90 structure; (**C**): surface representation of the HSP90 structure, including the selected grid box for molecular docking.

The HSP90 structure obtained through homology modeling based on the barnacle HSP90 homolog sequence (Figure 4) exhibits a typical chaperone arrangement dominated by *α*-helices and a few *β*-sheets. This modular architecture, consisting of an *N*-terminal domain (NTD), middle domain (MD), and *C*-terminal domain (CTD), is crucial for its function in protein folding, stabilization, and degradation [47]. Homology modeling has produced a protein structure using the protein with PDB ID 8ffw as a template (similarity: 81.99%), with Raman spectral analysis presented in Figure 4A. An assessment of the modeled structure reveals substantial alignment with theoretical expectations: a mere 1.3% of amino acid residues are situated in disallowed regions, whereas a staggering 89.9% are in the most favoured regions and 7.8% are in additional allowed regions, with a negligible 0.9% occupying generously acceptable regions. Ideally, a proportion of over 90% in the most favoured regions is preferred for high-confidence models; however, values in the range of 88–90% are generally acceptable for many biological studies [48]. Moreover, the model’s structural fidelity is reinforced by a Root Mean Square Deviation (RMSD) value of 1.303 across 373 atoms, as determined by overlaying the predicted structure with the template protein using PyMOL, underscoring a close structural congruence. Collectively, the structural soundness of the protein model is well founded and sufficiently robust to support its application in future research explorations.

### 2.6. Molecular Docking

**Figure 5:**
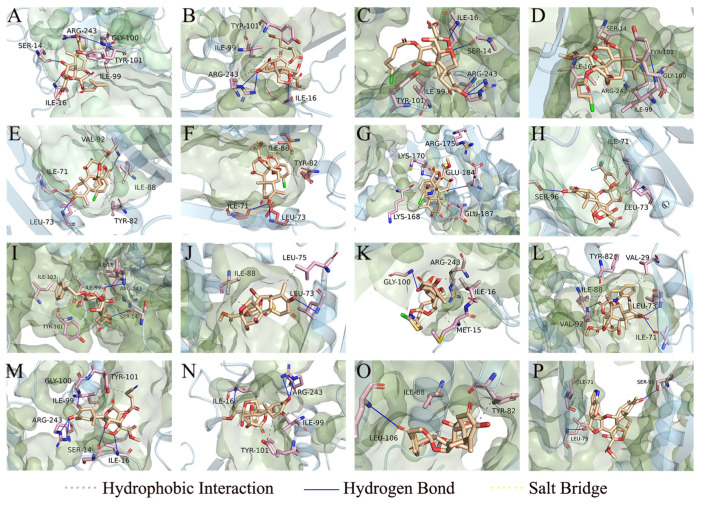
Schematic diagrams of the interaction mode between brusatol derivatives and the HSP90 molecule ((**A**–**P**) correspond to **B1**, **1**–**15**, respectively).

Molecular docking analyses demonstrated that compound **4** exhibited a relatively low binding energy (−6.71 kcal/mol) to HSP90 (Table 2, Figure 5), suggesting that it may achieve strong binding with HSP90 through interactions across multiple binding sites. Specifically, compound **4** forms hydrogen bonds with amino acids such as LEU73 and VAL92 and hydrophobic interactions with residues like TYR101 and ILE88, thereby further enhancing its binding affinity for HSP90. However, despite its low binding energy, the actual antifouling activity tests revealed that its EC_50_ value was 0.3527 µg/mL, with an LC_50_/EC_50_ ratio of only 2.1018, indicating that its efficacy and safety profile are not particularly remarkable. This discrepancy may stem from the inability of molecular docking simulations to adequately capture the complex and dynamic interactions between small molecules and proteins under physiological conditions [49]. While compound **4** forms strong bonds with the protein, this binding may not effectively block the protein’s critical functions, or the overly strong binding may trigger nonspecific effects, thereby compromising its overall bioactivity and selectivity [49]. In contrast, compound **3**, with a binding energy of −4.93 kcal/mol in molecular docking, exhibited superior antifouling efficacy (EC_50_ of 0.1475 µg/mL) and significantly higher safety (LC_50_/EC_50_ ratio of 42.2922) in bioactivity experiments, despite having a slightly higher binding energy than compound **4**. This phenomenon can be attributed to the formation of more stable hydrogen bonds and hydrophobic interactions between compound **3** and key amino acids in the protein, such as ARG243, TYR101, and ILE99. These amino acid residues are located near the active site of HSP90 and may induce conformational changes or interfere with protein–protein interactions, thereby disrupting the biological processes involved in barnacle attachment. Furthermore, the binding mode of compound **3** may be more adept at targeting specific functional domains of the protein, effectively blocking its activity while maintaining high selectivity, thereby achieving a more favorable balance between bioactivity and toxicity. Certainly, discrepancies between predicted binding energies from molecular docking and experimental bioactivity data are common, particularly when bioactivity values are close [49]. These differences arise from the simplifications inherent in docking algorithms, which may not capture small variations in activity effectively [49,50]. As a result, docking predictions often show weak or non-linear correlations with experimental outcomes [51]. While docking remains a valuable tool for predicting interactions, it does not always align perfectly with biological activity, especially for compounds with similar potencies [52]. In this study, different brusatol derivatives showed low binding energies. However, since their activity levels were relatively close (EC_50_ values ranging from 0.1475 to 4.3069 μg/mL), the differences in binding energies did not demonstrate a strict linear relationship with activity. Therefore, experimental validation is crucial. Integrating docking data with experimental bioactivity and toxicity results is essential for a more accurate and comprehensive understanding of the compounds’ mechanisms of action.

Key residues like ARG243, TYR101, and LEU73 may play a crucial role in stabilizing ligand binding to HSP90 through hydrophobic interactions and hydrogen bonds, as revealed by molecular docking studies of all the derivatives (Table 2). Strengthening these interactions by adding stronger hydrophobic groups or optimizing hydrogen bond donors and acceptors, such as derivatives **1**–**5**, could significantly increase the binding stability of small molecules in the HSP90 binding pocket. This conclusion aligns with previous research findings [53,54]. Similar previous studies indicate that boosting interactions with GLY97, ASP93, and THR184 enhances the specificity and potency of inhibitors binding to HSP90, resulting in improved therapeutic outcomes [53]. This suggests that different biological activities may specifically target various key amino acid sites on HSP90. Therefore, the optimization of small-molecule structures can be achieved by reinforcing interactions with key residues, accounting for protein flexibility, and utilizing molecular dynamics simulations. Structural modification guided by molecular docking enhances the derivatives’ inhibitory effects on HSP90 and offers clear guidance for future structure-based optimization and drug development. In conclusion, while molecular docking provides initial insights into protein–ligand interactions, combining it with bioactivity and toxicity data leads to more accurate predictions of small molecules’ real-world effects. This integrated approach helps guide the structural optimization of antifoulants, enhancing their potency and efficacy.

### 2.7. Structure–Activity Relationship Analysis

From the perspective of structure–activity relationships, various functional groups and side-chain structures significantly influence the activity and toxicity of compounds. For a clear understanding, the derivatives can be categorized into flexible-chain, benzene-ring, and heterocyclic side-chain derivatives. Flexible-chain derivatives generally show higher bioactivity compared to benzene-ring and heterocyclic derivatives. This may be due to the flexibility of the side chains, which allows them to fold more easily within the active pocket of HSP90, facilitating effective interaction between the active groups and the amino acids or other biological targets within the pocket [55,56]. Among the flexible-chain derivatives, compounds containing chlorine substitutions, such as compounds **2** and **3**, showed better activity and lower toxicity than those with bromine or cyano groups. This is because chlorine substituents are thought to enhance binding ability to biological targets, thus improving bioactivity. This observation is consistent with the literature, which indicates that electron-withdrawing groups like chlorine and bromine increase the hydrophobicity and lipophilicity of compounds, thereby boosting their activity [57,58,59,60,61]. Derivatives **3** and **2**, which contain chlorine substitutions, exhibited slightly higher activity than derivative **1**, which has bromine substitutions. This difference may be attributed to the greater electronegativity of chlorine compared to bromine, resulting in enhanced hydrophobicity and lipophilicity. This property facilitates the more effective interaction and binding of the compounds to the target protein [57,58,59,60,61]. Notably, compound **3**, with its shorter chain, displayed better activity than compound **2**. The shorter chains and simpler structures may contribute to the maintenance of strong activity and lower toxicity. For example, a study by Zhang et al. found that as the methylene chain lengthens, activity decreases, indicating that longer chains may diminish antifouling efficacy [62]. In support of this, it has been proposed that excessively long carbon chain structures can raise molecular hydrophobicity, which could lower activity in aqueous environments, thus reducing their antifouling effectiveness [63]. For benzene-ring derivatives, activity tends to decrease as the number of substituents on the ring increases. For instance, compound **4**, which contains only one chlorine substitution, exhibited the highest activity in this category, while compound **7**, with the most substitutions, showed the lowest activity. This further supports the idea that simpler core structures with fewer side chains result in better activity, aligning with the observations made earlier. This can also be attributed to the reduced steric hindrance of the flexible side chains, which allows them to more easily enter the protein’s active pocket and interact with the active site [55,56]. Similarly to the flexible-chain derivatives, chlorine-substituted benzene-ring derivatives tended to be more active than those containing fluorine or cyano groups. Some studies have demonstrated that introducing benzene rings, aromatic amino derivatives, or heterocycles such as pyridine rings can be beneficial for enhancing activity [63,64,65]. In the case of heterocyclic side chains, chlorine-substituted derivatives also showed better activity compared to bromine-substituted ones. Specifically, thiazole derivatives tended to outperform thiophene derivatives in terms of activity. Interestingly, an exception is noted in a bromine-substituted quinoline derivative, which, despite its relatively complex structure, exhibited the highest activity among the heterocyclic derivatives. This exception may be attributed to the common occurrence of individual variability in drug response in practical applications [66]. This suggests that in certain cases, a more complex heterocyclic structure can still achieve high activity, particularly when halogen substituents are present [42,44,45].

The structure–activity relationship analyses highlight that the selection and optimization of side-chain types, lengths, and functional group complexity have a significant impact on the bioactivity and toxicity of compounds. Shorter and simpler side chains generally lead to higher activity, while more complex structures tend to decrease it. The incorporation of chlorine atoms consistently enhances bioactivity while maintaining low toxicity. For example, derivatives with chlorine side chains, such as **3** and **6**, exhibited the best balance between activity and toxicity. Therefore, in future antifoulant design, focusing on simple side chains and chlorine incorporation is advisable. This strategy is likely to produce compounds that are both highly effective and exhibit low toxicity. Conversely, avoiding overly long or complex side chains will be crucial to minimize toxicity risks. This balanced strategy will guide the development of safer and more efficient antifoulants.

## 3. Materials and Methods

### 3.1. Plant Material

Brusatol was isolated and enriched from the dried fruit of *Brucea javanica* by Dr. Zhiwei Su, and the botanical fruits were identified by Dr. Zhonghui Ma. The voucher specimens (MZH20170896) are deposited in the Herbarium of Guangxi University’s College of Agriculture (GAUA).

### 3.2. General Chemicals

All reagents were acquired from commercial suppliers and used as received without additional purification. Thin-layer chromatography (TLC) was conducted on 0.25 mm thick precoated silica plates (Qingdao Puke Separation Materials Co., Ltd., Qingdao, China), with spot detection and visualization achieved using UV light at wavelengths of 365 and 254 nm. Chromatography on silica gel was performed using 300–400 mesh. Semi-preparative HPLC was carried out on a Shimadzu LC-2030C 3D Plus liquid chromatograph (YMC-Pack ODS-A columns, 10 × 250 mm) (Shimadzu Corporation, Kyoto, Japan). Organic extracts were dried over anhydrous magnesium sulfate and subsequently evaporated under reduced pressure at temperatures below 40 °C. High-resolution electrospray ionization mass spectrometry (ESI-MS) was conducted using a Waters Xevo G2-S QTOF/UPLC mass spectrometer (Waters Corporation, Milford, MA, USA). NMR spectroscopy was performed in CDCl_3_ (*δ*_H_ at 2.50 and 3.33 ppm; *δ*_C_ at 39.52 ppm) or C_5_D_5_N (*δ*_H_ at 7.22, 7.58, and 8.74 ppm; *δ*_C_ at 123.87, 135.91, and 150.35 ppm) (Cambridge Isotope Laboratories, Inc., Tewksbury, MA, USA) on a Bruker AVANCE III HD600 spectrometer (Bruker Corporation, Billerica, MA, USA) at Guangxi University, operating at 600 MHz for ^1^H and 150.9 MHz for ^13^C. The semi-synthesis of the target compounds was carried out at the Plant Systematics and Evolution Laboratory, College of Agriculture, Guangxi University, China.

### 3.3. General Synthesis Modification Procedures

#### 3.3.1. 3-*O*-(Trimethylsilyl)-Brusatol (**B2**)

To a solution of brusatol (**B1**, 200 mg, 0.384 mmol) in *N*, *N*-dimethylformamide (0.8 mL) were added t-butyldimethylchlorosilane (406 mg, 1.536 mmol) and imidazole (209 mg, 3.072 mmol), and the mixture was stirred at room temperature under a nitrogen atmosphere for 12 h. Subsequently, it was extracted with ethyl acetate and water, followed by recrystallization from ethanol and water to yield crude crystals. The precipitate was collected by filtration. These crude crystals were further purified by semi-preparative HPLC, using an acetonitrile/water (69:31) mixture as the eluent, to afford compound **B2** (212.0 mg, 0.3342 mmol, 87% yield) as a colorless amorphous solid. ^1^H NMR (600 MHz, Chloroform-*d*) *δ* 6.25 (s, 1H, H-15), 4.78 (s, 1H, H-7), 4.72 (d, *J* = 7.9 Hz, 1H, H-20 ax), 4.26 (d, *J* = 4.2 Hz, 1H, H-11), 4.19 (s, 1H, H-12), 3.79 (dd, *J* = 7.9 Hz, 1H, H-20 eq), 3.78 (s, 3H, 21-OCH_3_), 3.13 (s, 1H, H-14), 2.93 (d, *J* = 12.9 Hz, 1H, H-5), 2.89 (d, *J* = 15.8 Hz, 1H, H-1ax), 2.38 (dt, *J* = 14.7, 2.9 Hz, 1H, H-6 ax), 2.33 (d, *J* = 15.8 Hz, 1H, H-1eq), 2.18 (d, *J* = 1.3 Hz, 3H, 5′-CH_3_), 2.07 (*br* s, 1H, H-9), 1.92 (d, *J* = 1.4 Hz, 3H, 4′-CH_3_), 1.85 (d, *J* = 1.7 Hz, 3H, 18-CH_3_), 1.75 (ddd, *J* = 14.6, 13.0, 2.7 Hz, 1H, H-6 eq), 1.38 (d, *J* = 1.1 Hz, 3H, 19-CH_3_), 0.94 (s, 9H, H-Me_3_CSi), 0.17 (s, 3H, H-Me_2_Si), 0.13 (s, 3H, H-Me_2_Si). ^13^C NMR (150.9 MHz, Chloroform-*d*) *δ* 192.4 (C-2), 172.3 (C-21), 167.5 (C-16), 164.8 (C-1′), 161.3 (C-3′), 145.4 (C-3), 136.2 (C-4), 114.4 (C-2′), 82.9 (C-7), 81.7 (C-13), 76.1 (C-12), 74.4 (C-20), 71.3 (C-11), 66.1 (C-15), 53.4 (21-OCH_3_), 51.9 (C-14), 50.9 (C-1), 45.7 (C-8), 43.0 (C-5), 42.3 (C-9), 40.8 (C-10), 29.7 (C-6), 28.0 (C-4′), 26.4 (Me_3_CSi, 3C), 21.0 (C-5′), 19.2 (Me_3_CSi, C), 15.8 (C-19), 14.7 (C-18), −3.4 (Me_2_Si), −3.6 (Me_2_Si). MS (ESI): *m*/*z* = 635.39 [M+H]^+^, 652.42 [M+NH_4_]^+^, 657.38 [M+Na]^+^. Anal. calcd. for C_32_H_46_O_11_Si (634.28 g/mol).

#### 3.3.2. 3-*O*-(Trimethylsilyl)-Bruceolide (**B3**)

A mixture of compound **B2** (100 mg, 0.1577 mmol) in methyl alcohol (0.5 mL) was treated with sodium hydroxide (9.5 mg, 0.2366 mmol) in a 0 °C water bath. The mixture continued to be stirred at this temperature for 8 h. Upon completion, confirmed by TLC analysis, the reaction was halted. Any residual sodium hydroxide was neutralized with dilute hydrochloric acid to reach a neutral pH value. Subsequent steps involved extracting the mixture with ethyl acetate and water, followed by vacuum concentration. The crude product underwent silica gel column chromatography, utilizing an ethyl acetate–petroleum ether (7:3) mixture as the eluent, yielding compound **B3** (73.6 mg, 0.1332 mmol, 84% yield) as a colorless amorphous solid. ^1^H NMR (600 MHz, Chloroform-d) *δ* 5.28 (d, *J* = 14.7, 2.9 Hz 1H, H-15), 4.73 (s, 1H, H-7), 4.71 (d, *J* = 7.9 Hz, 1H, H-20 ax), 4.24 (*br* s, 1H, H-11), 4.21 (s, 1H, H-12), 3.78 (d, *J* = 7.9 Hz, 1H, H-20 eq), 3.78 (s, 3H, 21-OCH_3_), 3.03 (s, 1H, H-14), 2.86 (d, *J* = 12.9 Hz, 1H, H-5), 2.91 (d, *J* = 15.8 Hz, 1H, H-1ax), 2.38 (dt, *J* = 14.7, 2.9 Hz, 1H, H-6 ax), 2.28 (d, *J* = 15.8 Hz, 1H, H-1eq), 2.02 (d, *J* = 4.61 Hz, 1H, H-9), 1.85 (d, *J* = 1.7 Hz, 3H, 18-CH_3_), 1.77 (ddd, *J* = 14.6, 13.0, 2.7 Hz, 1H, H-6 eq), 1.39 (s, 3H, 19-CH_3_), 0.95 (s, 9H, H-Me_3_CSi), 0.17 (s, 3H, H-Me_2_Si), 0.14 (s, 3H, H-Me_2_Si). ^13^C NMR (150.9 MHz, Chloroform-*d*) *δ* 192.2 (C-2), 172.9 (C-21), 165.6 (C-16), 145.4 (C-3), 135.7 (C-4), 83.7 (C-7), 82.0 (C-13), 76.8 (C-12), 74.6 (C-20), 71.2 (C-11), 65.9 (C-15), 54.9 (21-OCH_3_), 53.6 (C-14), 51.0 (C-1), 45.6 (C-8), 43.2 (C-5), 43.1 (C-9), 40.7 (C-10), 29.7 (C-6), 26.4 (Me_3_CSi, 3C), 19.2 (Me_3_CSi, C), 15.8 (C-19), 14.7 (C-18), −3.4 (Me_2_Si), −3.6 (Me_2_Si). MS (ESI): *m*/*z* = 553.35 [M+H]^+^, 575.24 [M+Na]^+^, 591.22 [M+K]^+^. Anal. calcd. for C_27_H_40_O_10_Si (552.24 g/mol).

#### 3.3.3. 15-*O*-(5′-Bromovaleryl)-Bruceolide (**1**)

Into a solution of compound **B3** (50 mg, 0.0905 mmol) in dichloromethane (1 mL), 5-bromovaleryl chloride (27.1 mg, 0.1358 mmol), triethylamine (27.5 mg, 0.2715 mmol), and 4-dimethylaminopyridine (5.5 mg, 0.0453 mmol) were introduced. This mixture was stirred at room temperature for 8 h. Upon verification of completion by TLC analysis, the reaction mixture was concentrated via vacuum evaporation. It was then redissolved in tetrahydrofuran (1 mL), to which tetrabutylammonium fluoride (TBAF, 57.1 mg, 0.1810 mmol) was added, reacting for about 30 min to cleave the silyl protecting group from 3-OH, resulting in the formation of the desired compound **1**. The crude product was subsequently purified using silica gel column chromatography with an acetate–petroleum ether (3:2) eluent, leading to compound **1** (35.8 mg, 0.0597 mmol, 66% yield) as a colorless amorphous solid. ^1^H NMR (600 MHz, Chloroform-*d*) *δ* 6.36 (s, 1H, 12-OH), 6.10 (s, 1H, 11-OH), 4.75 (s, 1H, H-7), 4.73 (d, *J* = 7.9 Hz, 1H, H-20 ax), 4.23–4.26 (m, 1H, H-11), 4.19 (s, 1H, H-12), 3.84 (s, 3H, 21-OCH_3_), 3.79 (d, *J* = 7.9 Hz, 1H, H-20 eq), 3.56 (t, *J* = 6.0 Hz, 2H, H-5′), 3.38 (s, 1H, H-14), 3.04 (d, *J* = 12.9 Hz, 1H, H-5), 2.96 (d, *J* = 15.8 Hz, 1H, H-1 ax), 2.45–2.40 (m, 2H, H-2′), 2.38 (m, 2H, H-1eq, H-6 ax), 2.15 (*br* s, 1H, H-9), 1.84 (d, *J* = 1.7 Hz, 3H, 18-CH_3_), 1.83–1.80 (m, 4H, H-3′, H-4′), 1.73–1.80 (m, 1H, H-6 eq), 1.39 (d, *J* = 1.1 Hz, 3H, 19-CH_3_). ^13^C NMR (150.9 MHz, Chloroform-*d*) *δ* 192.2 (C-2), 172.3 (C-21), 171.5 (C-1′), 166.8 (C-16), 144.2 (C-3), 127.9 (C-4), 82.7 (C-7), 81.4 (C-13), 75.9 (C-12), 74.2 (C-20), 71.1 (C-11), 66.5 (C-15), 53.6 (21-OCH_3_), 53.3 (C-14), 48.7 (C-1), 45.6 (C-8), 44.6 (C-5′), 42.1 (C-5), 42.0 (C-9), 41.2 (C-10), 32.9 (C-2′), 31.6 (C-4′), 29.2 (C-6), 22.0 (C-3′), 15.6 (C-19), 13.5 (C-18). MS (ESI): *m*/*z* = 601.54 [M+H]^+^. Anal. calcd. for C_26_H_33_BrO_11_ (600.12 g/mol).

#### 3.3.4. 15-*O*-(5′-Chlorovaleryl)-Bruceolide (**2**)

For the synthesis of compound **2**, the same methodology employed for compound **1** was utilized. The acyl chloride involved in this reaction was 5-chlorovaleryl chloride. The crude product was subsequently purified using silica gel column chromatography with an acetate–petroleum ether (3:2) eluent, leading to compound **2** (32.1 mg, 0.0577 mmol, 64% yield) as a colorless amorphous solid. ^1^H NMR (600 MHz, Chloroform-*d*) *δ* 6.36 (s, 1H, OH), 6.10 (s, 1H, OH), 4.75 (s, 1H, H-7), 4.73 (d, *J* = 7.9 Hz, 1H, H-20 ax), 4.23–4.26 (m, 1H, H-11), 4.19 (s, 1H, H-12), 3.84 (s, 3H, 21-OCH_3_), 3.79 (d, *J* = 7.9 Hz, 1H, H-20 eq), 3.56 (t, *J* = 6.0 Hz, 2H, H-5′), 3.38 (s, 1H, H-14), 3.04 (d, *J* = 12.9 Hz, 1H, H-5), 2.96 (d, *J* = 15.8 Hz, 1H, H-1 ax), 2.45–2.40 (m, 2H, H-2′), 2.38 (m, 2H, H-1eq, H-6 ax), 2.15 (*br* s, 1H, H-9), 1.84 (d, *J* = 1.7 Hz, 3H, 18-CH_3_), 1.83–1.80 (m, 4H, H-3′, H-4′), 1.73–1.80 (m, 1H, H-6 eq), 1.39 (d, *J* = 1.1 Hz, 3H, 19-CH_3_). ^13^C NMR (150.9 MHz, Chloroform-*d*) *δ* 192.3 (C-2), 172.4 (C-21), 172.2 (C-1′), 166.9 (C-16), 144.2 (C-3), 128.0 (C-4), 82.7 (C-7), 81.4 (C-13), 75.9 (C-12), 74.1 (C-20), 71.1 (C-11), 66.5 (C-15), 53.3 (21-OCH_3_), 53.3 (C-14), 48.7 (C-1), 45.6 (C-8), 44.6 (C-5′), 42.0 (C-5), 42.0 (C-9), 41.2 (C-10), 32.9 (C-2′), 31.6 (C-4′), 29.2 (C-6), 21.9 (C-3′), 15.6 (C-19), 13.5 (C-18). MS (ESI): *m*/*z* = 557.20 [M+H]^+^, 579.21 [M+Na]^+^. Anal. calcd. for C_26_H_33_ClO_11_ (556.17 g/mol).

#### 3.3.5. 15-*O*-(4′-Chlorobutanoyl)-Bruceolide (**3**)

For the synthesis of compound **3**, the same methodology employed for compound **1** was utilized. The acyl chloride involved in this reaction was 4-chlorobutyryl chloride. The crude product was subsequently purified using silica gel column chromatography with an acetate–petroleum ether (3:2) eluent, leading to compound **3** (34.1 mg, 0.0629 mmol, 70% yield) as a colorless amorphous solid. ^1^H NMR (600 MHz, Chloroform-*d*) *δ* 6.12 (s, 1H, 11-OH), 4.75 (s, 1H, H-7), 4.72 (d, *J* = 7.9 Hz, 1H, H-20 ax), 4.23 (d, *J* = 4.37 Hz, 1H, H-11), 4.19 (s, 1H, H-12), 3.84 (s, 3H, 21-OCH_3_), 3.77 (d, *J* = 7.9 Hz, 1H, H-20 eq), 3.55 (t, *J* = 6.0 Hz, 2H, H-4′), 3.04 (s, 1H, H-14), 2.96 (d, *J* = 12.9 Hz, 1H, H-5), 2.95 (d, *J* = 15.8 Hz, 1H, H-1 ax), 2.42 (d, *J* = 15.8 Hz, 1H, H-1 eq), 2.37 (m, 1H, H-6 ax), 2.15 (*br* s, 1H, H-9), 1.83 (d, *J* = 1.7 Hz, 3H, 18-CH_3_), 1.83–1.80 (m, 4H, H-2′, H-3′), 1.75 (m, 1H, H-6 eq), 1.38 (d, *J* = 1.1 Hz, 3H, 19-CH_3_). ^13^C NMR (150.9 MHz, Chloroform-*d*) *δ* 192.2 (C-2), 172.3 (C-21), 171.5 (C-1′), 166.8 (C-16), 144.2 (C-3), 127.9 (C-4), 82.7 (C-7), 81.4 (C-13), 76.0 (C-12), 74.2 (C-20), 71.1 (C-11), 66.5 (C-15), 53.3 (21-OCH_3_), 53.3 (C-14), 48.7 (C-1), 45.6 (C-8), 44.6 (C-5′), 42.1 (C-5), 42.0 (C-9), 41.2 (C-10), 32.9 (C-2′), 31.6 (C-4′), 29.2 (C-6), 22.0 (C-3′), 15.6 (C-19), 13.5 (C-18). MS (ESI): *m*/*z* = 543.42 [M+H]^+^. Anal. calcd. for C_25_H_31_ClO_11_ (542.16 g/mol).

#### 3.3.6. 15-*O*-(5′-Chlorobenzoyl)-Bruceolide (**4**)

For the synthesis of compound **4**, the same methodology employed for compound **1** was utilized. The acyl chloride involved in this reaction was 4-chlorobenzoyl chloride. The crude product was subsequently purified using silica gel column chromatography with an acetate–petroleum ether (1:1) eluent, leading to compound **4** (34.5 mg, 0.0598 mmol, 66% yield) as a colorless amorphous solid. ^1^H NMR (600 MHz, pyridine-*d*_5_) *δ* 9.98 (s, 1H, 3-OH),8.12 (d, *J* = 8.4 Hz, 2H, H-4′, H-6′), 7.42 (d, *J* = 8.4 Hz, 2H, H-3′, H-7′), 6.60 (s, 1H, 12-OH), 5.17–5.13 (3H, overlapped, H-7, H-15, H-20 eq), 5.08 (s, 1H, H-12), 4.83 (d, *J* = 4.9 Hz, 1H, H-11), 4.01 (d, *J* = 7.9 Hz, 1H, H-20 ax), 4.13–4.08 (m, 1H, H-14), 3.61 (s, 3H, 21-OCH_3_), 3.31 (d, *J* = 15.8 Hz, 1H, H-1 ax), 3.07 (*br* s, 1H, H-5), 2.57–2.51 (m, 1H, H-1 eq), 2.36 (dt, *J* = 14.3, 3.3 Hz, 1H, H-6 ax), 2.69 (d, *J* = 4.61 Hz, 1H, H-9), 2.00 (d, *J* = 1.7 Hz, 3H, 18-CH_3_), 1.83–1.77 (m, 1H, H-6 eq), 1.66 (s, 3H, 19-CH_3_). ^13^C NMR (150.9 MHz, pyridine-*d*_5_) *δ* 193.4 (C-2), 171.7 (C-21), 168.4 (C-16), 164.3 (C-1′), 146.5 (C-3), 140.0 (C-5′), 132.1 (C-4′, C-6′), 129.5 (C-3′), 129.3 (C-7′), 128.6 (C-2′), 84.4 (C-7), 83.2 (C-13), 76.1 (C-12), 74.2 (C-20), 73.4 (C-11), 67.5 (C-15), 52.8 (21-OCH_3_), 50.4 (C-1), 46.7 (C-8), 42.8 (C-5), 42.7 (C-9), 41.7 (C-10), 30.4 (C-6), 16.1 (C-19), 13.8 (C-18). MS (ESI): *m*/*z* = 577.15 [M+H]^+^. Anal. calcd. for C_28_H_29_ClO_11_ (576.14 g/mol).

#### 3.3.7. 15-*O*-(5′-Chlorobenzoyl)-Bruceolide (**5**)

For the synthesis of compound **5**, the same methodology employed for compound **1** was utilized. The acyl chloride involved in this reaction was 5-chlorothiophene-2-carbonyl chloride. The crude product was subsequently purified using silica gel column chromatography with an acetate–petroleum ether (1:1) eluent, leading to compound **5** (37.4 mg, 0.0642 mmol, 71% yield) as a colorless amorphous solid. ^1^H NMR (600 MHz, Chloroform-*d*) *δ* 7.69 (d, *J* = 4.1 Hz, 1H, H-5′), 6.95 (dd, *J* = 4.1, 1.9 Hz, H-4′), 5.43 (t, *J* = 1.5 Hz, 1H, H-20 ax), 4.96 (d, *J* = 12.7 Hz, 1H, H-7), 4.84–4.80 (m, 1H, H-11), 4.77 (d, *J* = 7.9 Hz, 1H, H-20 ax), 4.29 (d, *J* = 4.9 Hz, 1H, H-12), 3.79 (d, *J* = 7.9 Hz, 1H, H-20 eq), 3.77 (s, 3H, 21-OCH_3_), 3.04 (d, *J* = 12.9 Hz, 1H, H-5), 2.95 (d, *J* = 15.8 Hz, 1H, H-1 ax), 2.89 (d, *J* = 12.78 Hz, 1H, H-14), 2.30 (d, *J* = 15.8 Hz, 1H, H-1 eq), 2.39 (d, *J* = 14.3 Hz, 1H, H-6 ax), 2.04 (s, 1H, H-9), 1.84 (d, *J* = 1.7 Hz, 3H, 18-CH_3_), 1.80 (m, 1H, H-6 eq), 1.40 (*br* s, 3H, 19-CH_3_). ^13^C NMR (150.9 MHz, Chloroform-*d*) *δ* 191.9 (C-2), 172.3 (C-21), 169.9 (C-16), 159.8 (C-1′), 144.2 (C-3), 139.3 (C-3′), 134.9 (C-6′), 130.3 (C-5′), 127.8 (C-4′), 128.1 (C-4), 83.4 (C-7), 81.3 (C-13), 76.2 (C-12), 73.8 (C-20), 69.5 (C-11), 65.8 (C-15), 53.6 (21-OCH_3_), 53.4 (C-14), 48.7 (C-1), 45.1 (C-8), 43.1 (C-5), 42.3 (C-9), 41.3 (C-10), 29.2 (C-6), 15.6 (C-19), 13.5 (C-18). MS (ESI): *m*/*z* = 583.14 [M+H]^+^, 605.10 [M+Na]^+^. Anal. calcd. for C_26_H_27_ClO_11_S (582.10 g/mol).

#### 3.3.8. 15-*O*-(3′-Chloropyridin-6′-Carbonyl)-Bruceolide (**6**)

For the synthesis of compound **6**, the same methodology employed for compound **1** was utilized. The acyl chloride involved in this reaction was 6-chloronicotinoyl chloride. The crude product was subsequently purified using silica gel column chromatography with an acetate–petroleum ether (1:1) eluent, leading to compound **6** (34.2 mg, 0.0593 mmol, 66% yield) as a colorless amorphous solid. ^1^H NMR (600 MHz, pyridine-*d*_5_) *δ* 9.17 (d, *J* = 2.4 Hz, 1H, H-3′), 8.29 (dd, *J* = 8.3, 2.4 Hz, 1H, H-7′), 7.42 (d, *J* = 8.3 Hz, 1H, H-6′), 5.19 (d, *J* = 2.8 Hz, 1H, H-15), 5.15 (d, *J* = 7.4 Hz, 1H, H-7), 5.07 (s, 1H, H-12), 4.84–4.81 (m, 1H, H-11), 4.11–4.07 (m, 1H, H-20 eq), 4.02 (d, *J* = 7.9 Hz, 1H, H-20 ax), 3.68 (s, 3H, 21-OCH_3_), 3.31 (d, *J* = 15.8 Hz, 1H, H-1 ax), 3.07 (*br* s, 1H, H-5), 2.55 (d, *J* = 15.8 Hz, 1H, H-1 eq), 2.37 (dt, *J* = 14.3, 3.3 Hz, 1H, H-6 ax), 2.71 (d, *J* = 4.61 Hz, 1H, H-9), 2.00 (d, *J* = 1.7 Hz, 3H, 18-CH_3_), 1.85–1.75 (m, 1H, H-6 eq), 1.66 (s, 3H, 19-CH_3_). ^13^C NMR (150.9 MHz, pyridine-*d*_5_) *δ* 193.4 (C-2), 171.7 (C-21), 168.2 (C-16), 167.6 (C-1′), 156.3 (C-5′), 151.9 (C-3′), 146.4 (C-3), 140.6 (C-7′), 125.7 (C-2′), 125.1 (C-6′), 84.5 (C-7), 83.3 (C-13), 76.1 (C-12), 74.2 (C-20), 73.4 (C-11), 66.0 (C-15), 52.9 (21-OCH_3_), 50.4 (C-1), 46.8 (C-8), 42.8 (C-5), 42.7 (C-9), 41.7 (C-10), 30.3 (C-6), 16.1 (C-19), 13.8 (C-18). MS (ESI): *m*/*z* = 578.15 [M+H]^+^. Anal. calcd. for C_27_H_28_ClNO_11_ (577.14 g/mol).

#### 3.3.9. 15-*O*-[5′-(Trifluoromethyl)-Benzoyl]-Bruceolide (**7**)

For the synthesis of compound **7**, the same methodology employed for compound **1** was utilized. The acyl chloride involved in this reaction was 4-(trifluoromethyl)benzoyl chloride. The crude product was subsequently purified using silica gel column chromatography with an acetate–petroleum ether (3:1) eluent, leading to compound **7** (35.5 mg, 0.0582 mmol, 64% yield) as a colorless amorphous solid. ^1^H NMR (600 MHz, pyridine-*d*_5_) *δ* 9.99 (s, 1H, 3-OH), 7.73 (overlapped, 2H, H-3′, H-7′), 7.64–7.54 (overlapped, 2H, H-4′, H-6′), 6.62 (s, 1H, 12-OH), 5.19–5.15 (2H, overlapped, H-7, H-15), 5.08 (s, 1H, H-12), 4.84 (s, 1H, H-11), 4.25–3.95 (3H, overlapped, H-14, H-20 ax, H-20 eq), 3.66 (s, 3H, 21-OCH_3_), 3.32 (d, *J* = 15.8 Hz, 1H, H-1 ax), 3.08 (*br* s, 1H, H-5), 2.55 (d, *J* = 15.8 Hz, 1H, H-1 eq), 2.37 (d, *J* = 14.3 Hz, 1H, H-6 ax), 2.75–2.64 (m, 1H, H-9), 1.99 (s, 3H, 18-CH_3_), 1.85–1.75 (m, 1H, H-6 eq), 1.67 (s, 3H, 19-CH_3_). ^13^C NMR (150.9 MHz, pyridine-*d*_5_) *δ* 193.4 (C-2), 171.7 (C-21), 168.3 (C-16), 146.4 (C-3), 134.7 (C-5′), 134.6 (C-3′), 134.1 (C-7′), 131.1 (C-2′), 126.2 (C-4′), 125.5 (C-6′), 125.1 (C-8′), 84.5 (C-7), 83.3 (C-13), 76.1 (C-12), 74.2 (C-20), 73.4 (C-11), 52.8 (21-OCH_3_), 50.4 (C-1), 46.8 (C-8), 42.8 (C-5), 42.8 (C-9), 41.7 (C-10), 30.4 (C-6), 16.1 (C-19), 13.8 (C-18). MS (ESI): *m*/*z* = 611.18 [M+H]^+^, 628.20 [M+NH_4_]^+^. Anal. calcd. for C_29_H_29_F_3_O_11_ (610.17 g/mol).

#### 3.3.10. 15-*O*-(5′-Fluorophenylacetyl)-Bruceolide (**8**)

3-fluorophenlacetic acid (20.9 mg, 0.1358 mmol), 1-(3-dimethylaminopropyl)-3-ethylcarbodiimide hydrochloride (EDCI, 34.7 mg, 0.1810 mmol), and triethylamine (27.5 mg, 0.2715 mmol) were dissolved in dichloromethane and stirred at room temperature for 2 h. TLC analysis indicated the formation of an activated intermediate via the reaction of the carboxylic acid with EDCI. Compound **B3** (50 mg, 0.0905 mmol) and 4-dimethylaminopyridine (5.5 mg, 0.0453 mmol) were then added, and the mixture was further reacted for 8 h. After TLC confirmed the completion of the reaction, the reaction mixture was concentrated under reduced pressure. The product was extracted with ethyl acetate and water. The organic layer was dried over anhydrous Na_2_SO_4_ and further concentrated under vacuum. TBAF (57.1 mg, 0.1810 mmol) in THF (0.5 mL) was added, and the mixture was stirred at room temperature for 0.5 h. Following another round of vacuum concentration and extraction using ethyl acetate and water, the target product was purified by silica gel column chromatography with an acetate–petroleum ether (2:1) eluent, leading to compound **8** (30.5 mg, 0.0532 mmol, 59% yield) as a colorless amorphous solid. ^1^H NMR (600 MHz, Chloroform-*d*) *δ* 8.13 (s, 1H, H-3′), 6.11 (s, 1H, 11-OH), 4.79 (s, 1H, H-7), 4.76 (d, *J* = 7.9 Hz, 1H, H-20 ax), 4.28 (d, *J* = 4.37 Hz, 1H, H-11), 4.21 (s, 1H, H-12), 3.83 (d, *J* = 7.9 Hz, 1H, H-20 eq), 3.79 (s, 3H, 21-OCH_3_), 3.25 (d, *J* = 12.78 Hz, 1H, H-14), 2.98 (d, *J* = 12.9 Hz, 1H, H-5), 2.97 (d, *J* = 15.8 Hz, 1H, H-1 ax), 2.46 (d, *J* = 15.8 Hz, 1H, H-1 eq), 2.46 (d, *J* = 14.3 Hz, 1H, H-6 ax), 2.22 (d, *J* = 3.90 Hz, 1H, H-9), 1.84 (d, *J* = 1.7 Hz, 3H, 18-CH_3_), 1.78 (*br* s, 1H, H-6 eq), 1.39 (*br* s, 3H, 19-CH_3_). ^13^C NMR (150.9 MHz, Chloroform-*d*) *δ* 192.1 (C-2), 172.1 (C-21), 169.6 (C-1′), 166.6 (C-16), 162.9 (C-5′, *J*_CF_ = 246.3 Hz), 144.2 (C-3), 135.2 (C-3′, *J*_CF_ = 8.41 Hz), 130.3 (C-7′, *J*_CF_ = 8.41 Hz), 127.8 (C-4), 125.3 (C-8′, *J*_CF_ = 2.92 Hz), 116.7 (C-4′, *J*_CF_ = 21.15 Hz), 114.5 (C-6′, *J*_CF_ = 21.15 Hz), 82.7 (C-7), 81.5 (C-13), 75.9 (C-12), 74.1 (C-20), 71.1 (C-11), 65.7 (C-15), 53.6 (21-OCH_3_), 53.2 (C-14), 48.7 (C-1), 45.7 (C-8), 42.1 (C-5), 42.0 (C-9), 41.2 (C-10), 40.2 (C-2′), 29.8 (C-6), 15.6 (C-19), 14.3 (C-18). MS (ESI): *m*/*z* = 597.23 [M+Na]^+^, 613.25 [M+K]^+^. Anal. calcd. for C_29_H_31_FO_11_ (574.16 g/mol).

#### 3.3.11. 15-*O*-(4′-Bromo-Thiophen-5′-Carbonyl)-Bruceolide (**9**)

For the synthesis of compound **9**, the same methodology employed for compound **8** was utilized, with the carboxylic acid involved in this reaction being 4-bromothiophene-3-carboxylic acid. Compound **9** was purified by silica gel column chromatography with an acetate–petroleum ether (2:1) eluent, leading to compound **9** (36.4 mg, 0.0582 mmol, 64% yield) as a colorless amorphous solid. ^1^H NMR (600 MHz, pyridine-*d*_5_) *δ* 9.93 (s, 1H, 3-OH), 8.49 (s, 1H, H-3′), 7.62 (d, *J* = 3.71 Hz, 1H, H-6′), 6.58 (s, 1H, 12-OH), 5.15–4.96 (overlapped, 4H, H-7, H-12, H-15, H-20 eq), 4.82 (d, *J* = 4.37 Hz, 1H, H-11), 4.09 (d, *J* = 12.56 Hz, 1H, H-14), 3.96 (d, *J* = 7.29 Hz, 1H, H-20 ax), 3.59 (s, 3H, 21-OCH_3_), 3.30 (d, *J* = 15.8 Hz, 1H, H-1 ax), 3.05 (d, *J* = 12.90 Hz, 1H, H-5), 2.54 (d, *J* = 14.3 Hz, 1H, H-6 ax), 2.33 (d, *J* = 15.8 Hz, 1H, H-1 eq), 2.65 (d, *J* = 4.61 Hz, 1H, H-9), 1.97 (d, *J* = 1.7 Hz, 3H, 18-CH_3_), 1.78 (m, 1H, H-6 eq), 1.64 (s, 3H, 19-CH_3_). ^13^C NMR (150.9 MHz, pyridine-*d*_5_) *δ* 193.4 (C-2), 171.6 (C-21), 168.3 (C-16), 160.8 (C-1′), 146.4 (C-3), 136.7 (C-5′), 130.9 (C-6′), 127.2 (C-3′), 111.7 (C-4′), 84.2 (C-7), 83.2 (C-13), 76.2 (C-12), 74.1 (C-20), 73.4 (C-11), 52.7 (21-OCH_3_), 50.4 (C-1), 46.6 (C-8), 42.8 (C-5), 42.6 (C-9), 41.7 (C-10), 29.9 (C-6), 16.1 (C-19), 13.8 (C-18). MS (ESI): *m*/*z* = 644.10 [M+NH_4_]^+^, 649.10 [M+Na]^+^. Anal. calcd. for C_26_H_27_BrO_11_S (626.05 g/mol).

#### 3.3.12. 15-*O*-(3′-Chloro-Thiazol-5′-Carbonyl)-Bruceolide (**10**)

For the synthesis of compound **10**, the same methodology employed for compound **8** was utilized, with the carboxylic acid involved in this reaction being 2-chloro-4-thiazolecarboxylic acid. Compound **10** was purified by silica gel column chromatography with an acetate–petroleum ether (2:1) eluent, leading to compound **10** (37.3 mg, 0.0639 mmol, 71% yield) as a colorless amorphous solid. ^1^H NMR (600 MHz, Chloroform-*d*) *δ* 8.13 (s, 1H, H-6′), 6.11 (s, 1H, 11-OH), 4.79 (s, 1H, H-7), 4.76 (d, *J* = 7.9 Hz, 1H, H-20 ax), 4.28 (d, *J* = 4.37 Hz, 1H, H-11), 4.21 (s, 1H, H-12), 3.83 (d, *J* = 7.9 Hz, 1H, H-20 eq), 3.79 (s, 3H, 21-OCH_3_), 3.25 (d, *J* = 12.78 Hz, 1H, H-14), 2.98 (d, *J* = 12.9 Hz, 1H, H-5), 2.97 (d, *J* = 15.8 Hz, 1H, H-1 ax), 2.46 (d, *J* = 15.8 Hz, 1H, H-1 eq), 2.46 (d, *J* = 14.3 Hz, 1H, H-6 ax), 2.22 (d, *J* = 4.61 Hz, 1H, H-9), 1.84 (d, *J* = 1.7 Hz, 3H, 18-CH_3_), 1.78 (*br* s, 1H, H-6 eq), 1.39 (*br* s, 3H, 19-CH_3_). ^13^C NMR (150.9 MHz, Chloroform-*d*) *δ* 192.2 (C-2), 172.0 (C-21), 166.3 (C-16), 158.6 (C-1′), 152.9 (C-3′), 144.2 (C-3), 144.0 (C-5′), 131.3 (C-6′), 127.9 (C-4), 83.0 (C-7), 81.4 (C-13), 75.8 (C-12), 74.2 (C-20), 71.1 (C-11), 67.4 (C-15), 53.6 (21-OCH_3_), 53.5 (C-14), 48.6 (C-1), 45.6 (C-8), 42.1 (C-5), 42.0 (C-9), 41.2 (C-10), 29.8 (C-6), 15.6 (C-19), 14.3 (C-18). MS (ESI): *m*/*z* = 584.18 [M+H]^+^, 606.09 [M+Na]^+^. Anal. calcd. for C_25_H_26_ClNO_11_S (583.09 g/mol).

#### 3.3.13. 15-*O*-(8′-Bromo-Quinolin-3′-Carbonyl)-Bruceolide (**11**)

For the synthesis of compound **11**, the same methodology employed for compound **8** was utilized, with the carboxylic acid involved in this reaction being 8-bromoquinoline-2-carboxylic acid. Compound **11** was purified by silica gel column chromatography with an acetate–petroleum ether (4:1) eluent, leading to compound **11** (33.7 mg, 0.0502 mmol, 55% yield) as a colorless amorphous solid. ^1^H NMR (600 MHz, pyridine-*d*_5_) *δ* 9.96 (s, 1H, 3-OH), 8.42 (d, *J* = 8.33 Hz, 1H, H-4′), 8.26 (d, *J* = 8.33 Hz, 1H, H-5′), 8.15 (dd, *J* = 7.42, 1.01 Hz, 1H, H-3′), 7.81 (dd, *J* = 8.33, 1.01 Hz, 1H, H-11′), 7.42 (t, *J* = 7.73 Hz, 1H, H-10′), 5.18–4.96 (overlapped, 4H, H-7, H-12, H-15, H-20 eq), 4.85 (d, *J* = 4.37 Hz, 1H, H-11), 4.21 (d, *J* = 12.56 Hz, 1H, H-14), 4.02 (d, *J* = 7.29 Hz, 1H, H-20 ax), 3.68 (s, 3H, 21-OCH_3_), 3.31 (d, *J* = 15.8 Hz, 1H, H-1 ax), 3.07 (*br* s Hz 1H, H-5), 2.71 (d, *J* = 4.61 Hz, 1H, H-9), 2.36 (d, *J* = 14.3 Hz, 1H, H-6 ax), 2.33 (d, *J* = 15.8 Hz, 1H, H-1 eq), 2.00 (d, *J* = 1.7 Hz, 3H, 18-CH_3_), 1.80 (m, 1H, H-6 eq), 1.67 (s, 3H, 19-CH_3_). ^13^C NMR (150.9 MHz, pyridine-*d*_5_) *δ* 193.4 (C-2), 171.8 (C-21), 168.2 (C-16), 164.2 (C-1′), 149.3 (C-3′), 146.4 (C-3), 145.4 (C-7′), 138.7 (C-5′), 134.9 (C-9′), 131.3 (C-6′), 129.8 (C-11′), 128.6 (C-4), 128.5 (C-9′), 126.7 (C-4′), 122.9 (C-7′), 84.4 (C-7), 83.2 (C-13), 76.2 (C-12), 74.2 (C-20), 73.4 (C-11), 53.0 (21-OCH_3_), 50.4 (C-1), 46.8 (C-8), 42.8 (C-5), 42.7 (C-9), 41.7 (C-10), 29.9 (C-6), 16.1 (C-19), 13.8 (C-18). MS (ESI): *m*/*z* = 672.12 [M+H]^+^, 689.12 [M+NH_4_]^+^, 694.19 [M+Na]^+^. Anal. calcd. for C_31_H_30_BrNO_11_ (671.10 g/mol).

#### 3.3.14. 15-*O*-(3′-Cyanopropanoyl)-Bruceolide (**12**)

Compound **B3** (50 mg, 0.0905 mmol), 3-cyanopropanoic acid (17.9 mg, 0.1810 mmol), *N*,*N*′-diisopropylcarbodiimide (DIC, 22.8 mg, 0.1810 mmol), 1-hydroxybenzotriazole (HOBT, 24.5 mg, 0.1810 mmol), and 4-dimethylaminopyridine (5.5 mg, 0.0453 mmol) were dissolved in dichloromethane and stirred at room temperature for 8 h. After the reaction, the mixture was concentrated under reduced pressure. The product was extracted sequentially with ethyl acetate/10% HCl, ethyl acetate/saturated NaHCO_3_, and ethyl acetate/water. The organic layer was dried over anhydrous Na_2_SO_4_ and then further concentrated under vacuum. TBAF (57.1 mg, 0.1810 mmol) in THF (0.5 mL) was added, and the mixture was stirred at room temperature for 0.5 h. Following another round of vacuum concentration and extraction using ethyl acetate and water, the target product was purified by silica gel column chromatography with an acetate–petroleum ether (1:1) eluent, leading to compound **12** (33.8 mg, 0.0652 mmol, 72% yield) as a colorless amorphous solid. ^1^H NMR (600 MHz, Chloroform-*d*) *δ* 6.10 (s, 1H, 11-OH), 4.77 (s, 1H, H-7), 4.74 (d, *J* = 7.9 Hz, 1H, H-20 ax), 4.25 (*br* s, 1H, H-11), 4.21 (s, 1H, H-12), 3.79 (d, *J* = 7.9 Hz, 1H, H-20 eq), 3.81 (s, 3H, 21-OCH_3_), 3.05 (d, *J* = 12.78 Hz, 1H, H-14), 2.96 (d, *J* = 15.80 Hz, 1H, H-1 ax), 2.94 (d, *J* = 12.90 Hz, 1H, H-5), 2.75 (m, 1H, H-3′), 2.68 (m, 1H, H-2′), 2.46 (d, *J* = 14.3 Hz, 1H, H-6 ax), 2.44 (d, *J* = 15.80 Hz, 1H, H-1 eq), 2.17 (m, 1H, H-9), 1.85 (d, *J* = 1.7 Hz, 3H, 18-CH_3_), 1.78 (m, 1H, H-6 eq), 1.39 (s, 3H, 19-CH_3_). ^13^C NMR (150.9 MHz, Chloroform-*d*) *δ* 192.2 (C-2), 174.1 (C-21), 168.5 (C-16), 166.5 (C-1′), 144.2 (C-3), 129.0 (C-4), 118.5 (C-4′), 83.3 (C-7), 81.6 (C-13), 75.9 (C-12), 74.1 (C-20), 73.9 (C-11), 65.7 (C-15), 53.6 (21-OCH_3_), 53.5 (C-14), 48.6 (C-1), 45.8 (C-8), 42.2 (C-5), 42.0 (C-9), 41.2 (C-10), 32.1 (C-2′), 29.8 (C-6), 15.6 (C-19), 14.3 (C-18), 13.0 (C-3′). MS (ESI): *m*/*z* = 520.18 [M+H]^+^, 542.18 [M+Na]^+^, 537.22 [M+NH_4_]^+^. Anal. calcd. for C_25_H_29_NO_11_ (519.17 g/mol).

#### 3.3.15. 15-*O*-(5′-Cyano-Phenyl-3′-Acetyl)-Bruceolide (**13**)

For the synthesis of compound **13**, the same methodology employed for compound **12** was utilized, with the carboxylic acid involved in this reaction being 3-cyanobenzeneacetic acid. Compound **13** was purified by silica gel column chromatography with an acetate–petroleum ether (2:1) eluent, leading to compound **13** (37.0 mg, 0.0636 mmol, 70% yield) as a colorless amorphous solid. ^1^H NMR (600 MHz, pyridine-*d*_5_) *δ* 9.93 (s, 1H, 3-OH), 8.02 (d, *J* = 8.31 Hz, 1H, H-6′), 7.71 (*br* s, 1H, H-4′), 7.51 (d, *J* = 7.37 Hz, 1H, H-7′), 7.26 (d, *J* = 7.73 Hz, 1H, H-8′), *δ* 6.59 (s, 1H, 12-OH), 5.14–4.96 (overlapped, 4H, H-7, H-12, H-15, H-20 eq), 4.78 (d, *J* = 4.37 Hz, 1H, H-11), 3.94 (d, *J* = 7.29 Hz, 1H, H-20 ax), 3.83 (s, 3H, 21-OCH_3_), 3.75 (*br* s, 1H, H-2′), 3.27 (d, *J* = 15.8 Hz, 1H, H-1 ax), 3.02 (*br* s Hz 1H, H-5), 2.60 (d, *J* = 4.61 Hz, 1H, H-9), 2.50 (d, *J* = 14.3 Hz, 1H, H-6 ax), 2.30 (d, *J* = 15.8 Hz, 1H, H-1 eq), 1.96 (d, *J* = 1.7 Hz, 3H, 18-CH_3_), 1.76 (m, 1H, H-6 eq), 1.63 (s, 3H, 19-CH_3_). ^13^C NMR (150.9 MHz, pyridine-*d*_5_) *δ* 193.4 (C-2), 171.8 (C-21), 168.2 (C-16), 168.3 (C-1′), 146.4 (C-3), 134.9 (C-3′), 133.9 (C-4′), 131.3 (C-8′), 129.9 (C-6′), 139.6 (C-7′), 128.6 (C-4), 119.5 (C-9′), 113.0 (C-5′), 84.3 (C-7), 83.3 (C-13), 76.2 (C-12), 74.1 (C-20), 73.4 (C-11), 52.8 (21-CH_3_), 50.4 (C-1), 46.6 (C-8), 42.7 (C-5), 42.5 (C-9), 41.7 (C-10), 40.4 (C-2′), 29.9 (C-6), 16.0 (C-19), 13.8 (C-18). MS (ESI): *m*/*z* = 582.08 [M+H]^+^, 635.29 [M+H]^+^. Anal. calcd. for C_30_H_31_NO_11_ (581.19 g/mol).

#### 3.3.16. 15-*O*-(3′-Acetyl-Thiophen-6′-Carbonyl)-Bruceolide (**14**)

For the synthesis of compound **14**, the same methodology employed for compound **12** was utilized, with the carboxylic acid involved in this reaction being 5-acetylthiophene-2-carboxylicacid. Compound **14** was purified by silica gel column chromatography with an acetate–petroleum ether (3:1) eluent, leading to compound **14** (34.6 mg, 0.0587 mmol, 65% yield) as a colorless amorphous solid. ^1^H NMR (600 MHz, Chloroform-*d*) *δ* 7.83 (d, *J* = 4.04 Hz, 1H, H-5′), 7.64 (d, *J* = 4.10 Hz, 1H, H-4′), 4.83 (s, 1H, H-7), 4.76 (d, *J* = 7.90 Hz, 1H, H-20 ax), 4.29 (d, *J* = 4.37 Hz, 1H, H-11), 4.22 (s, 1H, H-12), 3.85 (d, *J* = 7.90 Hz, 1H, H-20 eq), 3.73 (s, 3H, 21-OCH_3_), 3.20 (d, *J* = 12.78 Hz, 1H, H-14), 2.99 (d, *J* = 15.80 Hz, 1H, H-1 ax), 2.98 (d, *J* = 12.90 Hz, 1H, H-5), 2.60 (s, 3H, 8′-CH_3_) 2.45 (d, *J* = 15.80 Hz, 1H, H-1 eq), 2.42 (d, *J* = 14.3 Hz, 1H, H-6 ax), 2.18 (d, *J* = 4.61 Hz, 1H, H-9), 1.86 (d, *J* = 1.7 Hz, 3H, 18-CH_3_), 1.78 (ddd, *J* = 15.40, 13.10, 2.80 Hz, 1H, H-6 eq), 1.42 (s, 3H, 19-CH_3_). ^13^C NMR (150.9 MHz, Chloroform-*d*) *δ* 192.0 (C-2), 190.9 (C-7′), 172.0 (C-21), 166.2 (C-16), 160.2 (C-1′), 152.9 (C-5′), 150.0 (C-3′), 144.2 (C-3), 137.5 (C-6′), 135.1 (C-5), 131.9 (C-4′), 127.6 (C-4), 82.9 (C-7), 81.5 (C-13), 75.9 (C-12), 74.2 (C-20), 71.1 (C-11), 65.3 (C-15), 53.6 (21-OCH_3_), 48.7 (C-1), 45.9 (C-8), 42.2 (C-5), 42.0 (C-9), 41.3 (C-10), 29.8 (C-6), 27.1 (C-8′), 15.6 (C-19), 13.5 (C-18). MS (ESI): *m*/*z* = 591.16 [M+H]^+^, 608.18 [M+NH_4_]^+^, 613.15 [M+Na]^+^. Anal. calcd. for C_28_H_30_O_12_S (590.15 g/mol).

#### 3.3.17. 15-*O*-(Indole-3′-Carbonyl)-Bruceolide (**15**)

1*H*-indol-4-amine (33.5 mg, 0.2534 mmol), carbonyldiimidazole (41.1 mg, 0.2534 mmol), triethylamine (36.6 mg, 0.3620 mmol), and 4-dimethylaminopyridine (4.4 mg, 0.0362 mmol) were dissolved in acetonitrile (1 mL) and stirred for 2 h to form an intermediate product. Compound **B3** (50 mg, 0.0905 mmol) was then added, and the mixture was stirred for an additional 6 h. After the reaction’s completion was confirmed by TLC, the reaction mixture was concentrated under reduced pressure. The product was extracted with ethyl acetate and water. The organic phase was dried over anhydrous Na_2_SO_4_ and then further concentrated under vacuum. TBAF (57.1 mg, 0.1810 mmol) in THF (0.5 mL) was added, and the mixture was stirred at room temperature for 0.5 h. Following another round of vacuum concentration and extraction using ethyl acetate and water, the target product was purified by silica gel column chromatography with an acetate–petroleum ether (3:1) eluent, leading to compound **15** (33.4 mg, 0.0576 mmol, 64% yield) as a colorless amorphous solid. ^1^H NMR (600 MHz, pyridine-*d*_5_) *δ* 12.89 (s, 1H, N-H), 9.66 (s, 1H, 3-OH), 9.02 (d, *J* = 1.43 Hz, H-5′), 8.43 (dd, *J* = 8.52, 1.43 Hz, 1H, H-7′), 7.52 (*br* s, 1H, H-8′), 7.41 (d, *J* = 8.52 Hz, 1H, H-3′), 6.57 (*br* s, 1H, 12-OH), 6.47 (*br* s, 1H, H-4′), 5.9 (s, 1H, 11-OH), 5.14–4.96 (overlapped, 5H, H-7, H-12, H-11, H-15, H-20 eq), 3.94 (d, *J* = 7.29 Hz, 1H, H-20 ax), 3.69 (s, 3H, 21-OCH_3_), 3.29 (d, *J* = 15.8 Hz, 1H, H-1 ax), 3.20 (*br* s, 1H, H-5), 2.75 (d, *J* = 4.61 Hz, 1H, H-9), 2.59 (d, *J* = 14.3 Hz, 1H, H-6 ax), 2.30 (d, *J* = 15.8 Hz, 1H, H-1 eq), 1.97 (d, *J* = 1.7 Hz, 3H, 18-CH_3_), 1.79 (m, 1H, H-6 eq), 1.70 (s, 3H, 19-CH_3_). ^13^C NMR (150.9 MHz, pyridine-*d*_5_) *δ* 193.4 (C-2), 173.9 (C-1′), 171.4 (C-21), 167.8 (C-16), 146.3 (C-3), 140.3 (C-10′), 128.8 (C-4), 128.6 (C-9′), 127.7 (C-6′), 125.1 (C-3′), 122.0 (C-5′), 112.2 (C-7′, C-8′), 103.8 (C-4′), 83.9 (C-7), 82.2 (C-13), 77.6 (C-12), 74.7 (C-20), 70.3 (C-11), 52.8 (21-OCH_3_), 50.2 (C-1), 45.9 (C-8), 43.2 (C-5), 42.8 (C-9), 41.9 (C-10), 30.3 (C-6), 16.3 (C-19), 14.1 (C-18). MS (ESI): *m*/*z* = 608.18 [M+NH_4_]^+^, 613.16 [M+Na]^+^. Anal. calcd. for C_30_H_31_NO_11_ (581.19 g/mol).

### 3.4. Barnacle Culture

#### 3.4.1. Collection and Cultivation of Mature Barnacles

Mature *Balanus amphitrite* were predominantly harvested from mangrove branches that are densely covered with these organisms, located along the coastal mudflats of Shanxin Village in Fangchenggang, Guangxi, China (108°11′12.32″ E, 21°34′45.97″ N). The cultivation process was meticulously conducted under controlled laboratory conditions, with slight modifications to existing methods [46,67,68,69]. The artificial seawater salinity was maintained at about 3.0%, the same as that of Beibu Gulf in Guangxi, and the temperature was kept steady at 28 °C using a constant-temperature seawater heater, which is especially important during the autumn and the winter. The seawater’s oxygenation was ensured through an oxygen pump, complemented by a lighting regime that simulated a natural 12 h day cycle.

Feeding procedures employed a dual approach, utilizing both animal-derived *Artemia nauplii* larvae and plant-based *Phaeodactylum tricornutum* Bohlin as nutritional sources. The barnacles were provided with frozen *A. nauplii* larvae, introduced into each glass tank to achieve a density of about 200 individuals per liter. Additionally, 5 L of freshly cultured algal solution was diluted into 30 L of seawater. The protocol included a routine of water replacement and feeding every three days, ensuring optimal conditions for the barnacles’ growth and well-being.

#### 3.4.2. Collection and Metamorphic Cultivation of Barnacle Larvae

In the hours between 9 AM and 12 PM, barnacle larvae were coaxed into clustering by a half-hour exposure to the intense beam of a handheld flashlight, serving as a focused light source. These clustered larvae were subsequently gathered into a 500 mL beaker. Four 40-L transparent glass tanks were readied, each filled with previously sterilized seawater (the salinity was meticulously kept between 29% and 30%, with a temperature range of 28 to 29 °C). To this setup, 3 L of a rich, dark brown algal solution were added to 24–25 L of the sterilized seawater, complemented by the introduction of 6 mL of potassium penicillin (at a concentration of 87.5 g/L) and 6 mL of streptomycin (50 g/L), to prevent bacterial contamination. A small oxygen pump gently aerated the water, ensuring an optimal oxygen level. The barnacle nauplii, once collected, were transferred into these tanks to be reared, with water changes scheduled every two days and daily observations made to monitor larval development. After roughly four days of such meticulous care, some of the stage VI nauplii began their transformation into cyprids. These cyprid larvae were then carefully collected into pristine Petri dishes for further observation. The dishes were preserved in a refrigerator, maintained at a chilly 2 to 4 °C overnight, and readied for subsequent study or experimentation.

### 3.5. Anti-Settlement Assays

Compounds dissolved in DMSO were sequentially administered to the wells, starting with a concentration of 25 µg/mL and proceeding with serial half-logarithmic dilutions. The volume of the compound in each well was maintained at 5 µg, with seawater as the blank control, DMSO as the negative control, and SeaNine 211 serving as the positive control. Utilizing a stereo microscope, a 1 mL micropipette was employed to introduce 995 μL of seawater and between 21 and 25 cyprid larvae into each well of a 24-well plate. Subsequently, the plates were incubated in a darkened, temperature-controlled environment for 48 h. After incubation, microscopic examination revealed the count of deceased and adhered cyprid larvae. This observation facilitated the calculation of mortality and settlement rates of the barnacle cyprids, crucial for assessing the antifouling properties of the compounds. The determination of EC_50_ values for the inhibition of barnacle larval attachment and LC_50_ values for larval mortality offered an evaluation of the compounds’ efficacy in deterring barnacle larval adherence and their associated toxicity [46].

### 3.6. Rescue Assay of Balanus Amphitrite Cyprid Larvae

A resuscitation assay was performed to assess the recovery ability of *Balanus amphitrite* cyprid larvae following drug treatment, as documented by Han Zhuang [46]. Derivatives (compounds **1**–**4**, **6**, **8**, **10**, **11**) with potent antifouling activity were selected, as these compounds demonstrated significantly higher efficacy than brusatol (*p* < 0.001). A concentration of 1 μg/mL was chosen because it allowed for high larval survival without attachment, facilitating parallel comparisons across derivatives. Cyprid larvae were exposed to each compound at 1 μg/mL for 24 h. Surviving, unattached larvae were rinsed with filtered seawater and transferred to 24-well plates containing 1 mL of filtered seawater. Settlement was monitored after 48 h, and the resuscitation rate was determined by the attachment rate.

### 3.7. Zebrafish Embryo Toxicity Assay

The zebrafish embryo toxicity assay was conducted according to the method described by Huang et al. [70], with slight modifications. Test compounds were dissolved in 0.5% DMSO to prepare a concentration series (0.1, 1, 5, 25, 50 μg/mL), which was then dispensed into 24-well plates. Fifteen cleavage-stage embryos were added to each well. The plates were incubated at 28 °C, and embryonic development was evaluated under a stereomicroscope after 48 h of exposure.

### 3.8. Homologous Modeling of HSP90

The original HSP90 sequence from patterned barnacle larvae was obtained from a literature database [71]. To predict the protein model, the HSP90 sequence was obtained through a Blast search on the UniProt website “https://www.uniprot.org/blast” (accessed on 18 July 2024). The primary sequence of the domain-containing protein was then used for model construction via the Swiss Model server “https://swissmodel.expasy.org/” (accessed on 18 July 2024). The 3D structure of heat shock protein HSP90-alpha (PDB: 8ffw) was selected as the most appropriate template, exhibiting an 81.99% sequence identity [29]. The quality of the Swiss model was evaluated by generating a Ramachandran plot using Procheck software UCLA-DOE LAB-Saves v6.1 “https://saves.mbi.ucla.edu/” (accessed on 18 July 2024). Finally, images of the predicted model were created using PyMOL. Protein–ligand docking software Auto Vina v1.5.6 was utilized to examine the binding energies and interactions among candidate small molecules and their targets.

### 3.9. Ligand and Receptor Preparation for Molecular Docking Analyses

This research utilized Autodock version 1.5.6 to facilitate this process. Files containing proteins and ligands were restructured into PDBQT format, with water molecules removed and polar hydrogens added for detailed analysis. The docking area was strategically placed at the central axis to capture the essential structural domains of the proteins, ensuring that molecules had the freedom to move without constraints. The grid box position for the binding site was determined by referencing the active site of the small-molecule ligand in the template protein. The precise coordinates are center_x = 109.481, center_y = 115.141, and center_z = 146.42, with dimensions of size_x = 46, size_y = 44, and size_z = 36. Visualization of the molecular docking interactions was achieved through PyMOL v2.5.4 software.

## Figures and Tables

**Table 1:** Anti-larval settlement activities of newly synthesized brusatol derivatives against *Balanus amphitrite*.

Derivatives	*Balanus amphitrite*		
	EC_50_ (μg/mL)	LC_50_ (μg/mL)	LC_50_/EC_50_
**B1**	2.7826 ± 0.6874	56.1254 ± 5.2154	20.1701
**1**	0.5235 ± 0.1229	0.8418 ± 0.1123	1.5564
**2**	0.4169 ± 0.1851	1.1247 ± 0.1442	2.6978
**3**	0.1475 ± 0.0332	6.2381 ± 2.6188	42.2922
**4**	0.3527 ± 0.1009	0.7413 ± 0.0820	2.1018
**5**	2.0248 ± 0.2374	6.1412 ± 0.1728	3.0330
**6**	0.3402 ± 0.1043	5.7709 ± 2.5367	16.9633
**7**	2.9678 ± 0.3430	6.9359 ± 0.8115	2.3371
**8**	1.1205 ± 0.1033	5.9076 ± 1.0251	5.2723
**9**	2.8392 ± 0.2415	5.7608 ± 0.8267	2.0290
**10**	0.6423 ± 0.0593	1.0139 ± 0.0923	1.5785
**11**	0.3274 ± 0.1845	1.1398 ± 0.1794	3.4814
**12**	1.3858 ± 0.1349	2.8020 ± 0.1225	2.0219
**13**	1.8426 ± 0.1791	6.6065 ± 0.1123	3.5854
**14**	2.1452 ± 0.7272	7.9213 ± 0.2432	3.6926
**15**	4.3069 ± 0.3275	6.7964 ± 0.5721	1.5780
SeaNine 211 *	1.9363 ± 0.4128	33.9306 ± 2.0625	31.9943

* Positive control.

**Table 2:** Interaction results of ligands docked with HSP90 molecule.

Ligand	Binding Energy (kcal/mol)	Interacting Residues	
		Hydrophobic Interactions	Hydrogen Bonds
**B1**	−5.20	ILE99, TYR101, ARG243	SER14, ILE16, GLY100, ARG243
**1**	−5.02	ILE99, TYR101, ARG243	ILE1, ARG243
**2**	−4.77	ILE99, TYR101, ARG243	SER14, ILE16, ARG243
**3**	−4.93	SER14, ILE16, GLY100, ARG243	ILE16, ILE99, TYR101, ARG243
**4**	−6.71	LEU73, TYR82, ILE88	ILE71, LEU73, VAL92
**5**	−6.08	LEU73, TYR82, ILE88	ILE71, LEU73
**6**	−6.46	LYS168, LYS170, GLU187	LYS170, GLU184
**7**	−5.56	ILE71, LEU73	LEU73, SER96
**8**	−5.47	SER14, SER14	ILE16, ILE99, TYR101, ILE103, AGR243
**9**	−5.88	LEU73, LEU75, ILE88	LEU73
**10**	−6.44	MET15, ILE16	GLY100
**11**	−6.60	VAL29, TYR82, ILE88	ILE71, LEU73, VAL92
**12**	−4.88	ILE16, ILE99, TYR101, ARG243	SER14, ILE16, GLY100, ARG243
**13**	−5.93	ILE99, TYR101, ARG243	ILE16, ARG243
**14**	−5.72	TYR82, ILE88	LEU106
**15**	−6.50	ILE71, LEU73	LEU73, SER96
SeaNine 211 *	−3.35	ILE170, GLU184, PHE188	LYS170

* Positive control.

## Data Availability

Data are contained within the article.

## Supplementary Materials

The following supporting information can be downloaded at https://www.mdpi.com/article/10.3390/ijms26020593/s1.
