# Supplementary material for: New Brusatol Derivatives as Anti-Settlement Agents Against Barnacles, Targeting HSP90: Design, Synthesis, Biological Evaluation, and Molecular Docking Investigations
Source: Int J Mol Sci. 2025 Jan 12;26(2):593. doi: 10.3390/ijms26020593 (PMC11765156; doi:10.3390/ijms26020593)
Supplement: Supplementary file 1 [file ijms-26-00593-s001.zip › ijms-3371797-supplementary.pdf]

Supporting Information

New Brusatol Derivatives as Anti-Settlement Agents Against Barnacles, Targeting HSP90: Design, Synthesis, Biological Evaluation, and Molecular Docking Investigations

Wang Jiang[1, 2, 3], Tongtong Luan[3], Pei Cao[3], Zhonghui Ma[1, 2], Zhiwei Su[1, 2, 3]*

[1]College of Agriculture, Guangxi University, Nanning 530004, China

[2]Traditional Chinese Herbal Medicine Resources and Agriculturalization Research Institute, Guangxi University, Nanning 530004, China

[3]Institute of Marine Drugs, Guangxi University of Chinese Medicine, Nanning 530200, China

* Correspondence: suzhiwei@gxu.edu.cn; Tel.: +86-188-7888-4206.

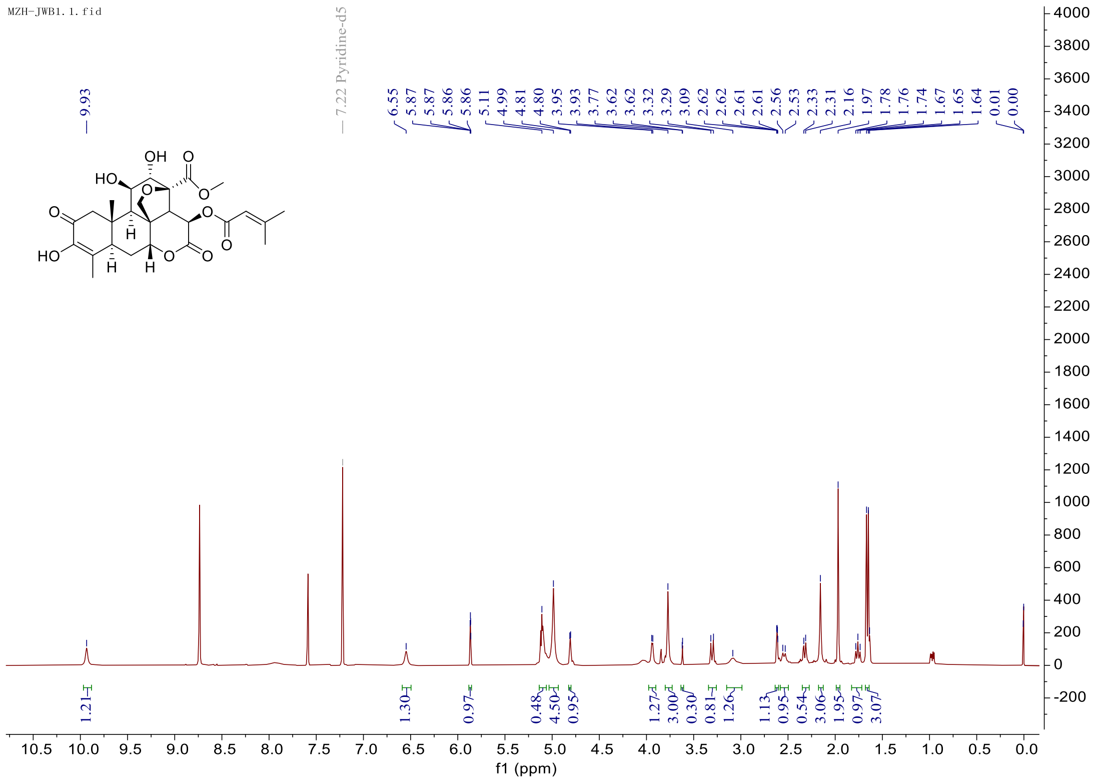

Figure S1. ^{1}H NMR spectrum of brusatol (**B1**)

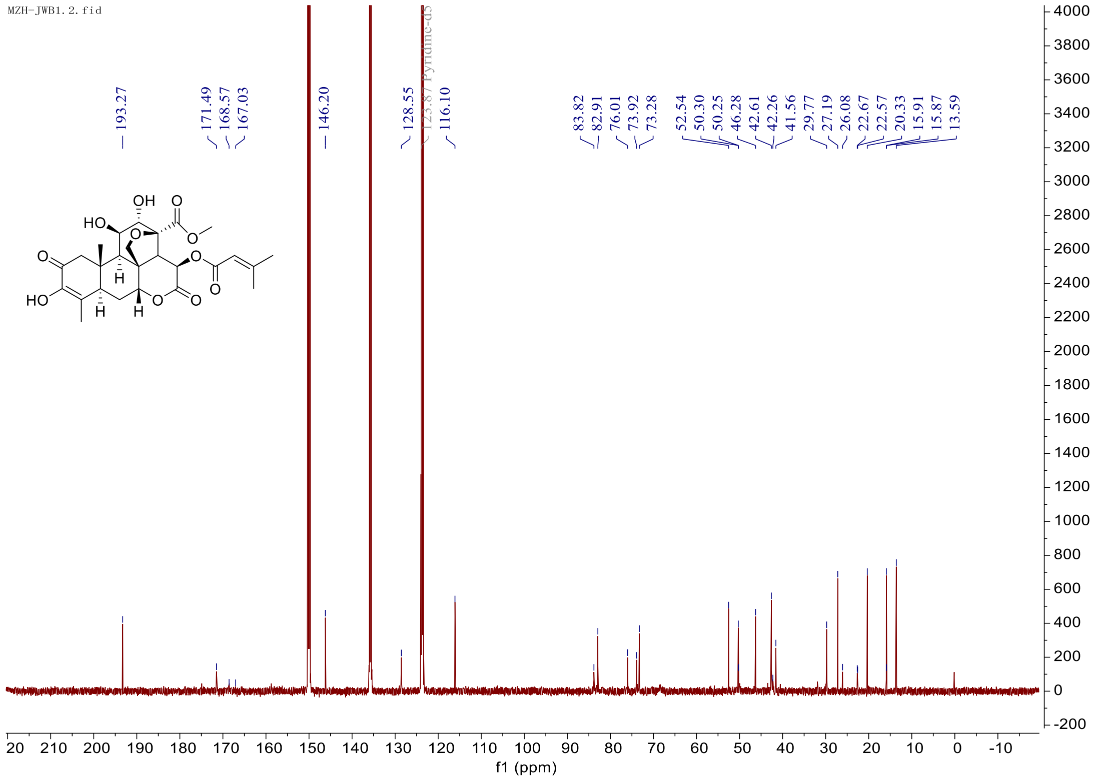

Figure S2. ^{13}C NMR spectrum of brusatol (**B1**)

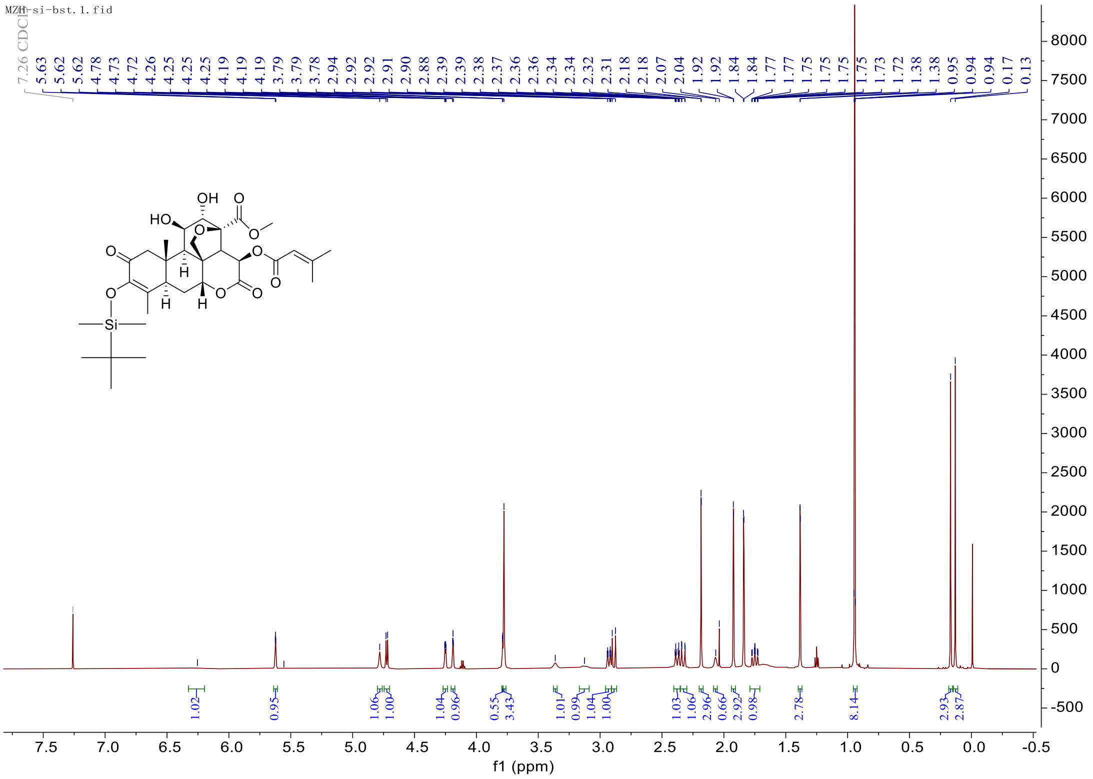

Figure S3. ^{1}H NMR spectrum of compound **B2**

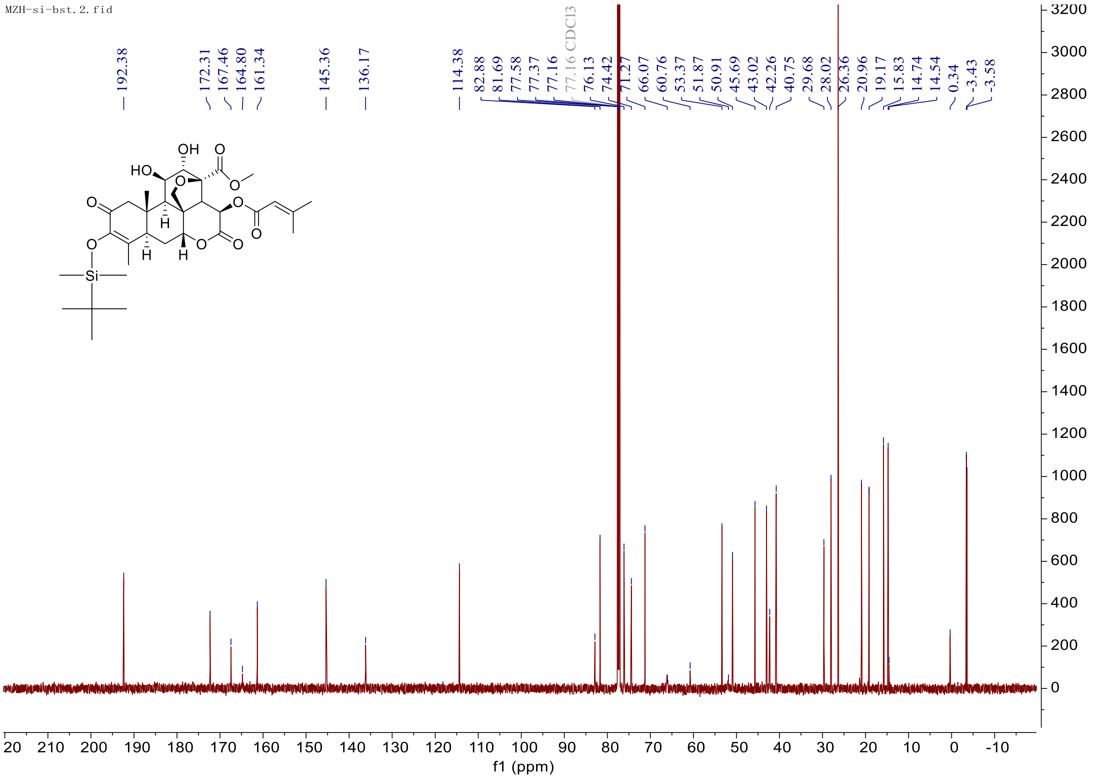

Figure S4. ^{13}C NMR spectrum of compound **B2**

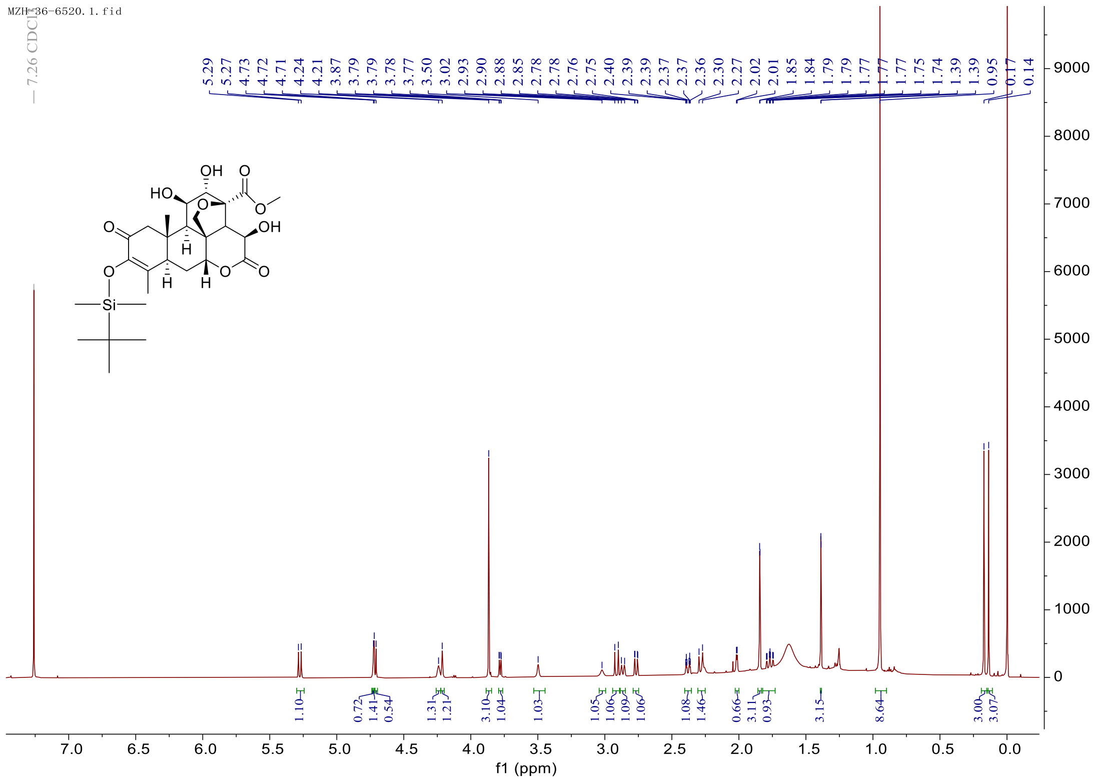

Figure S5. ^{1}H NMR spectrum of compound **B3**

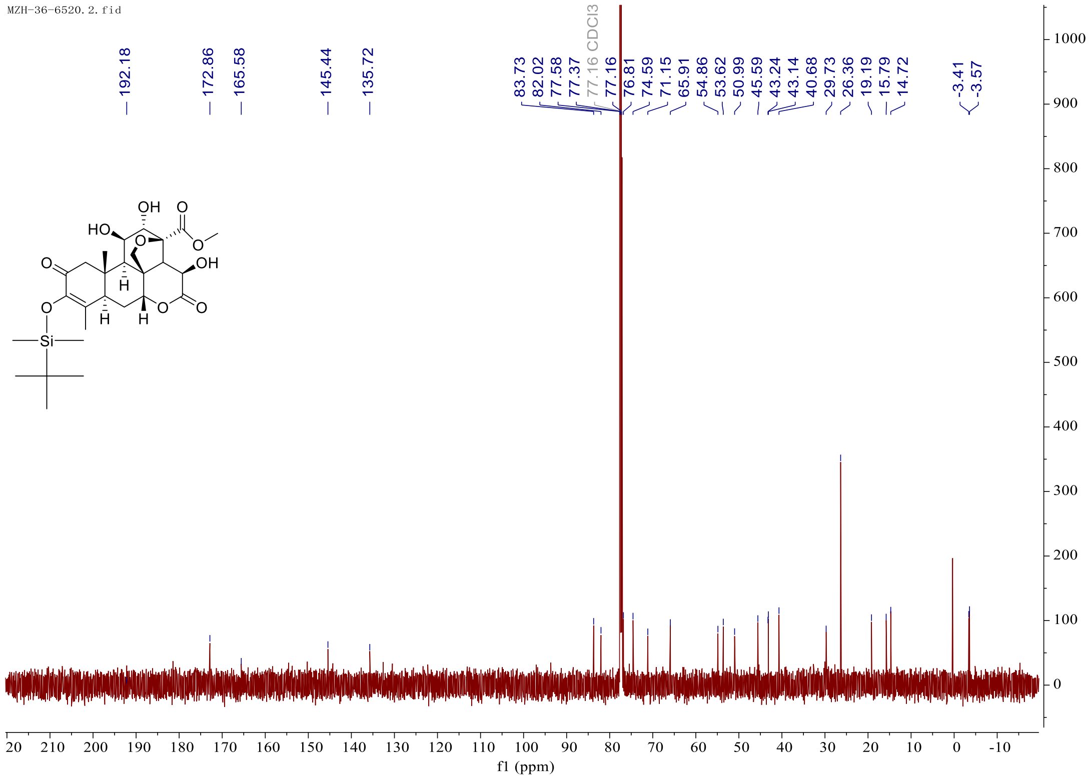

Figure S6. ^{13}C NMR spectrum of compound **B3**

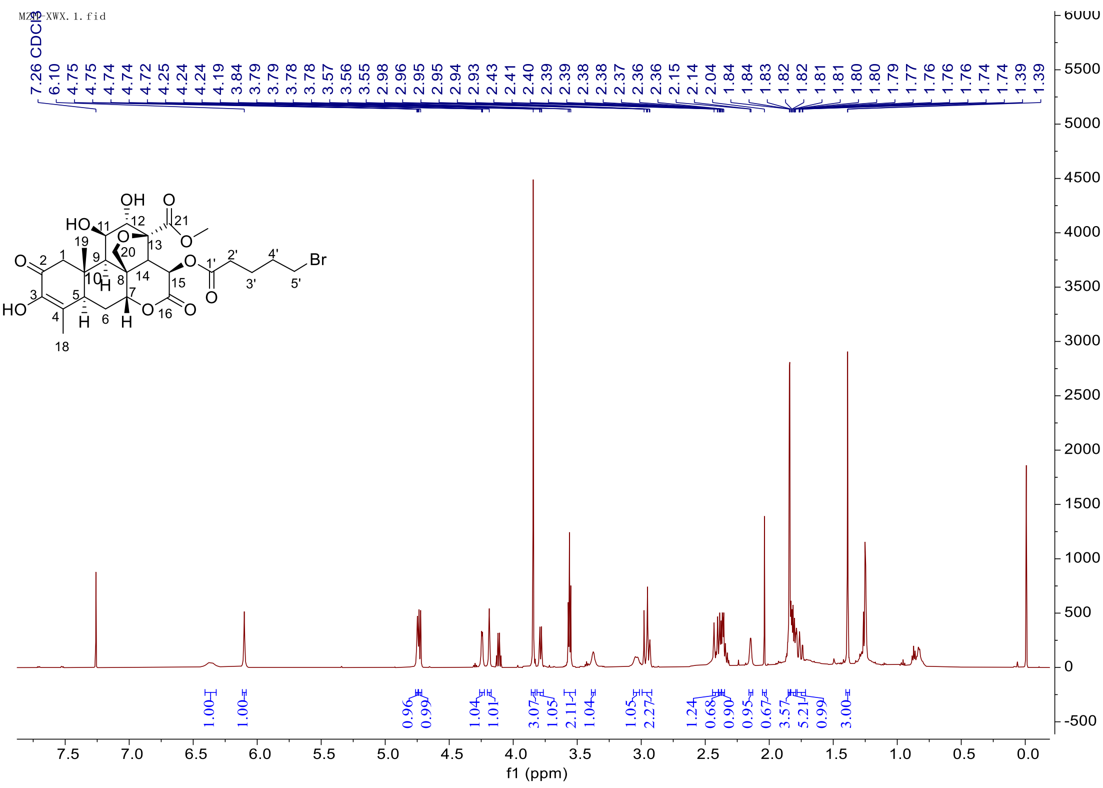

Figure S7. ^{1}H NMR spectrum of compound **1**

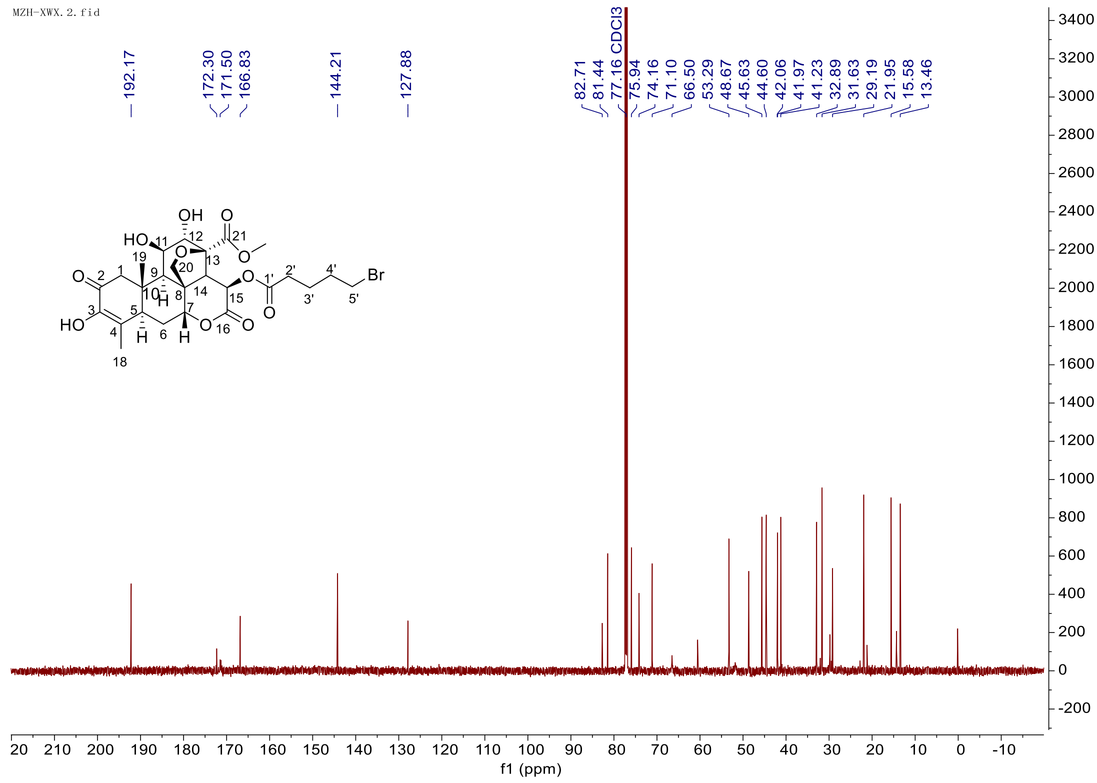

Figure S8. ^{13}C NMR spectrum of compound **1**

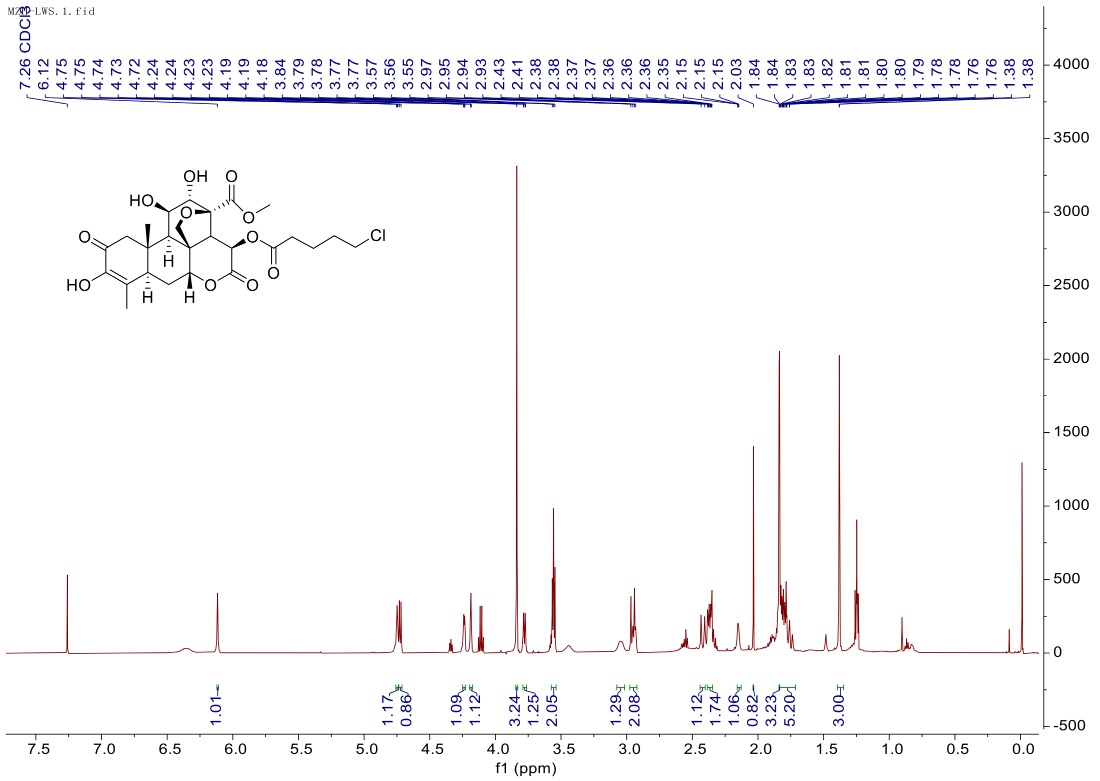

Figure S9. ^{1}H NMR spectrum of compound **2**

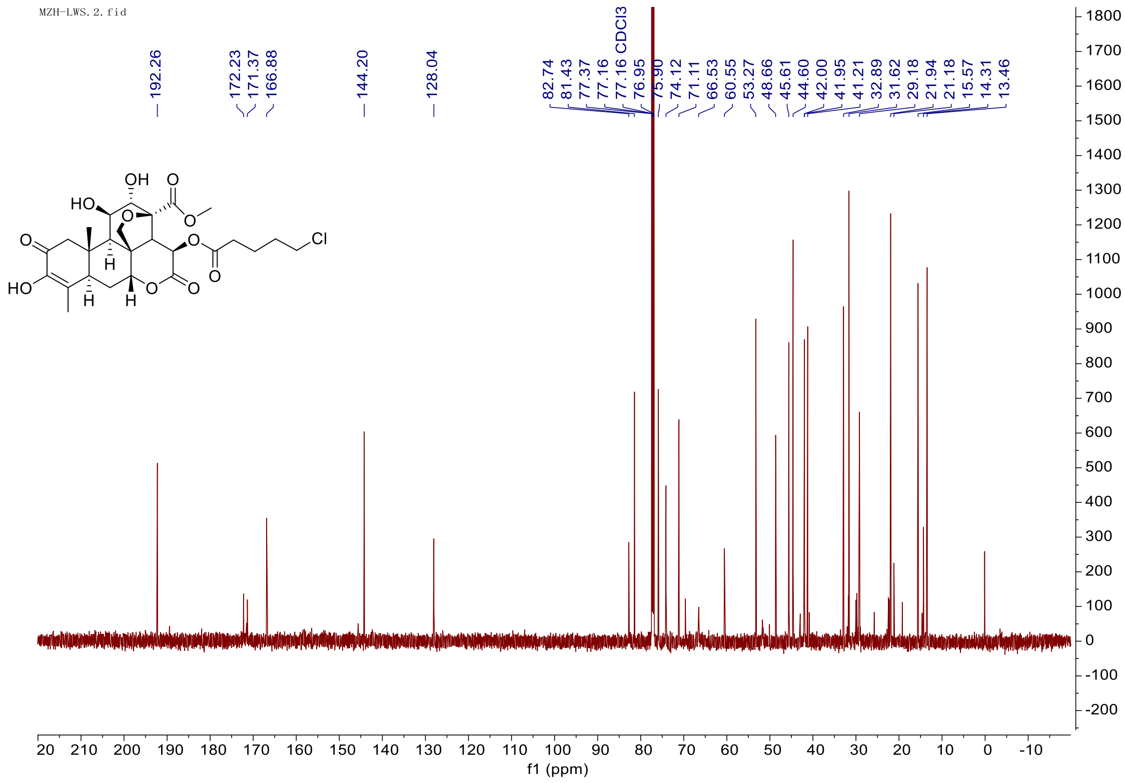

Figure S10. ^{13}C NMR spectrum of compound **2**

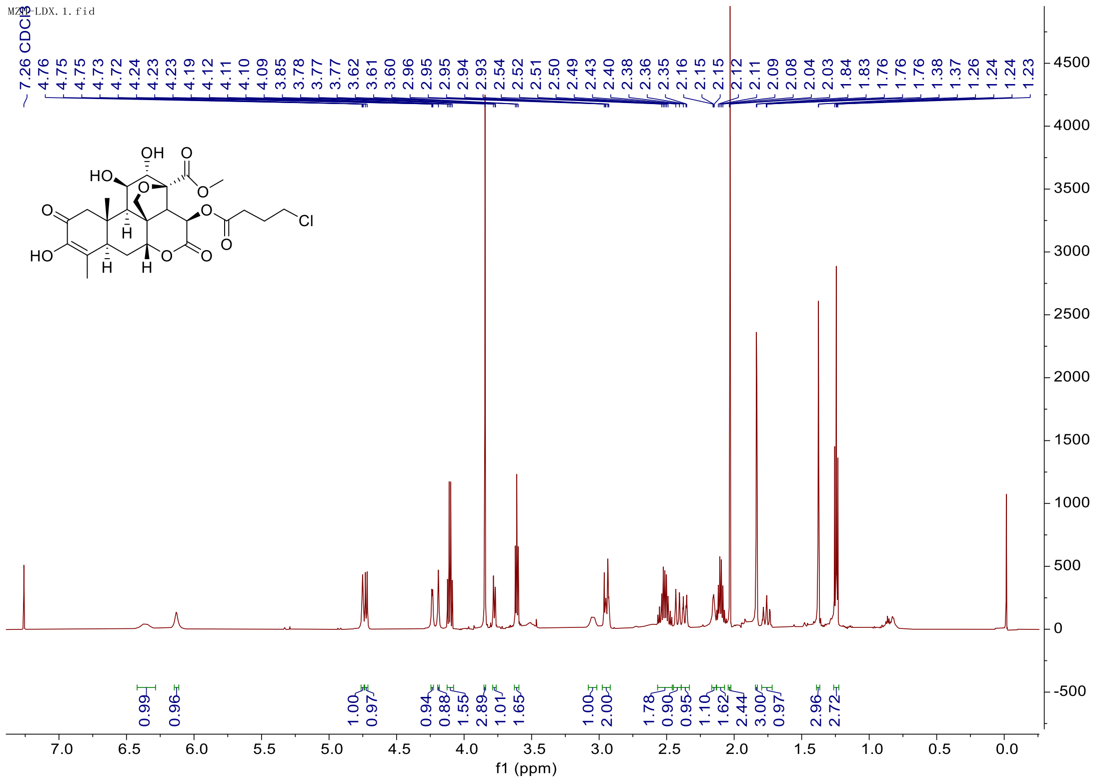

Figure S11. ^{1}H NMR spectrum of compound **3**

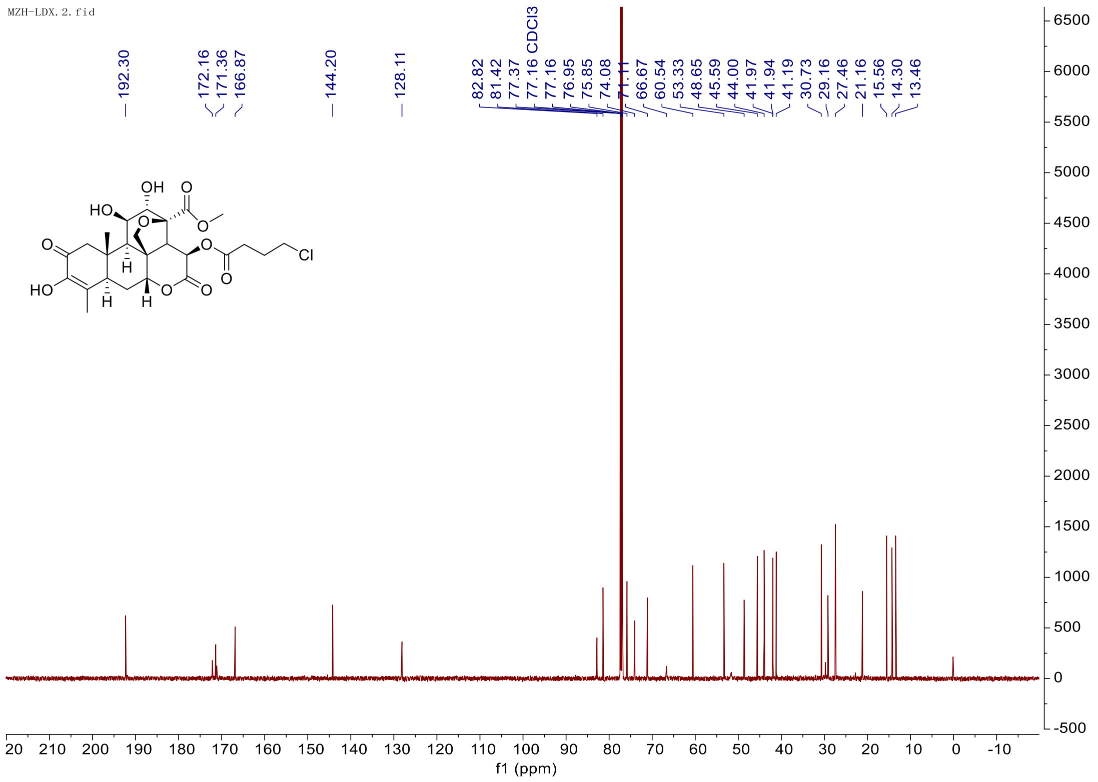

Figure S12. ^{13}C NMR spectrum of compound **3**

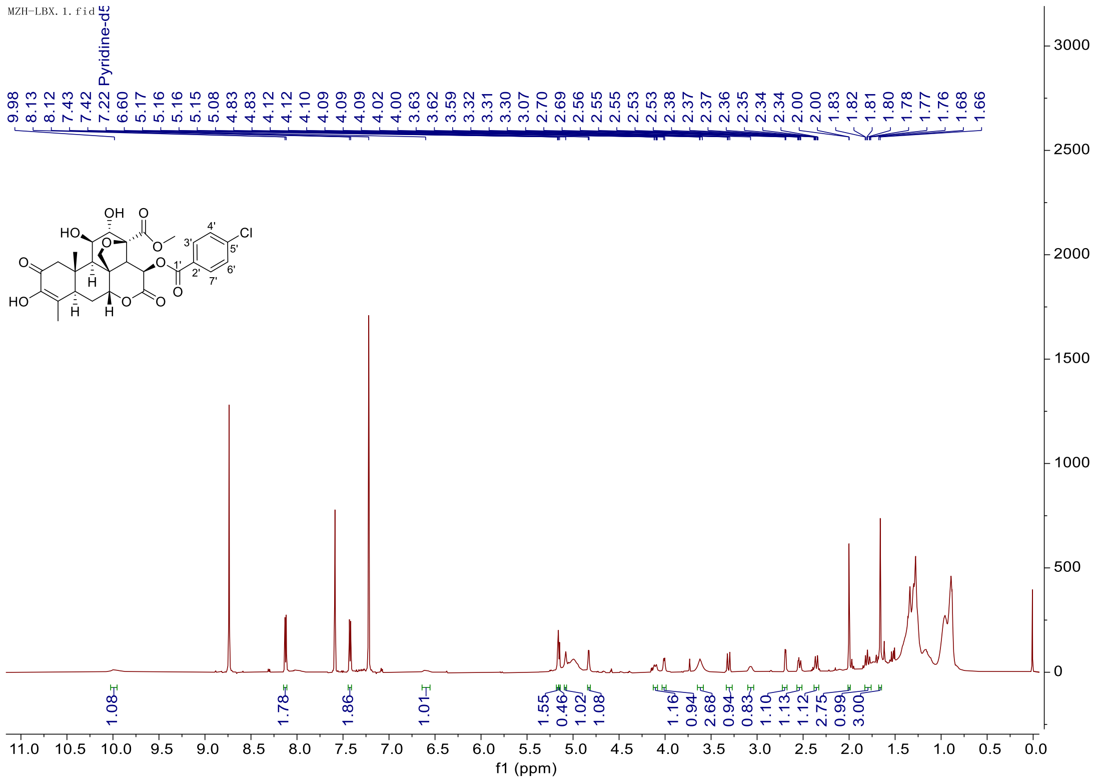

Figure S13. ^{1}H NMR spectrum of compound **4**

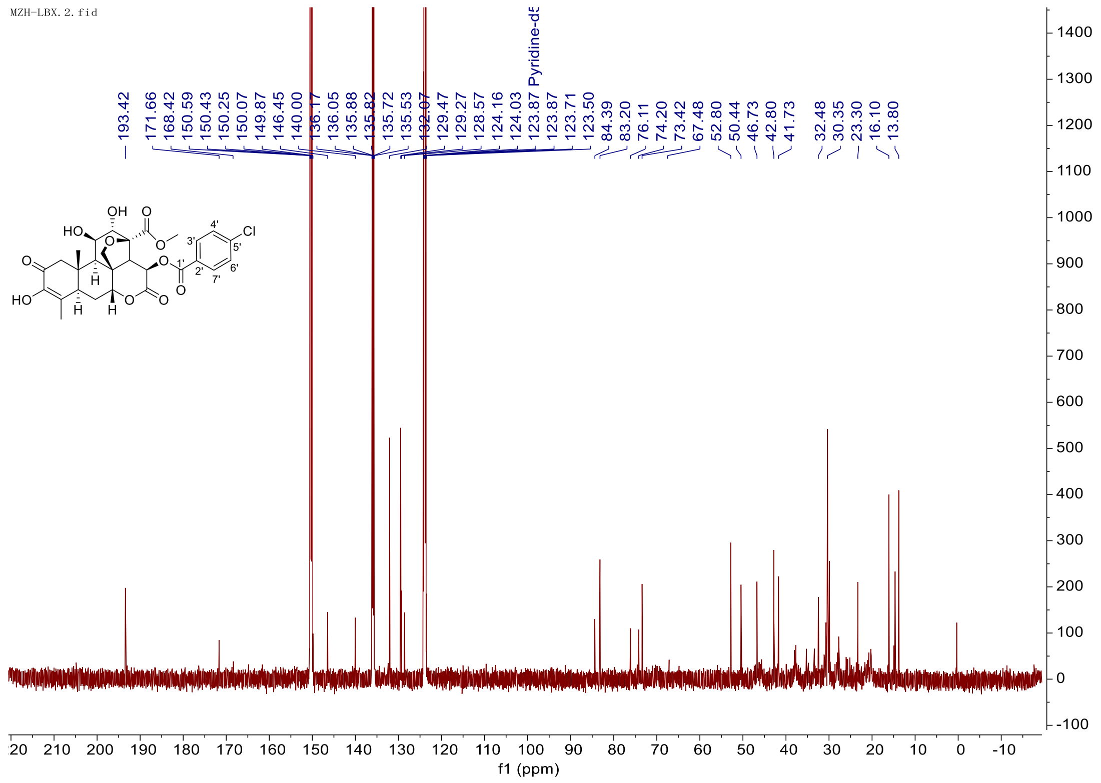

Figure S14. ^{13}C NMR spectrum of compound **4**

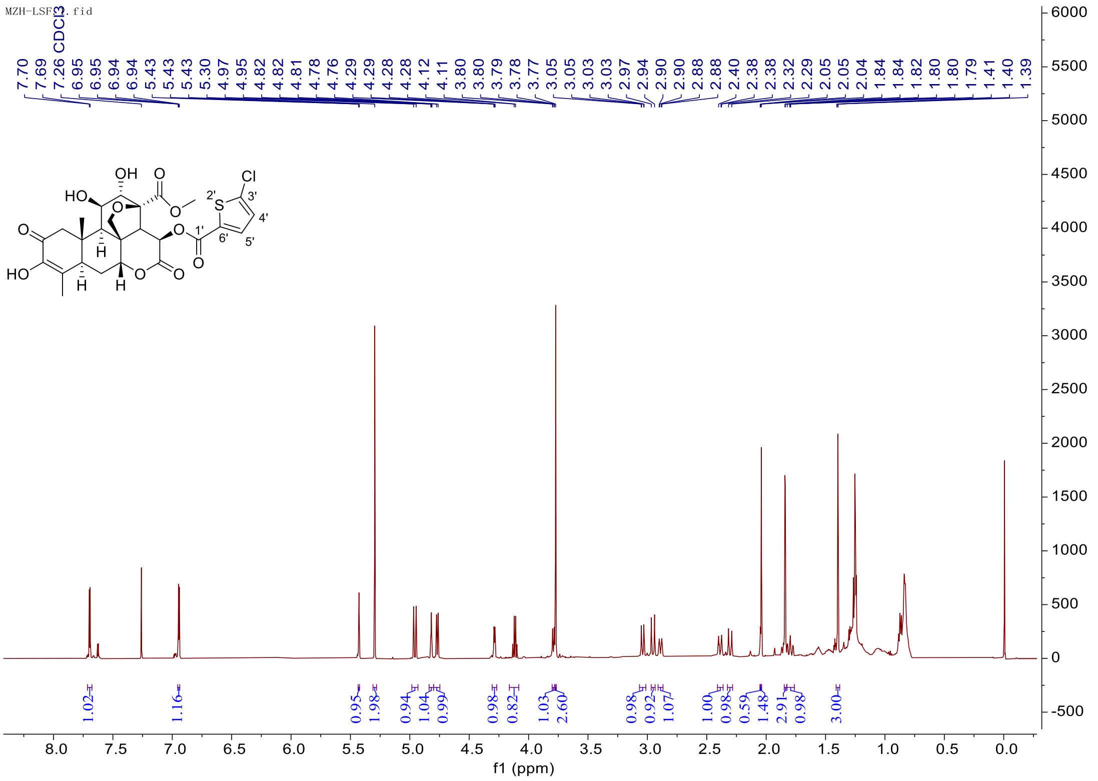

Figure S15. ^{1}H NMR spectrum of compound **5**

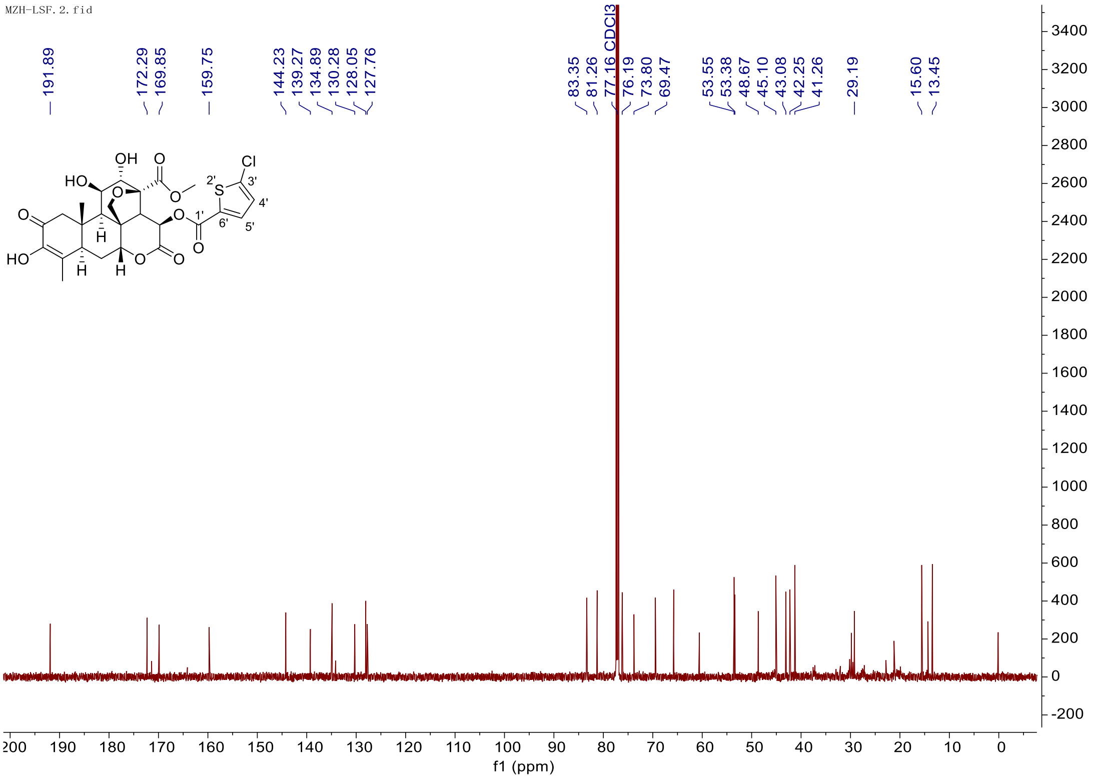

Figure S16. ^{13}C NMR spectrum of compound **5**

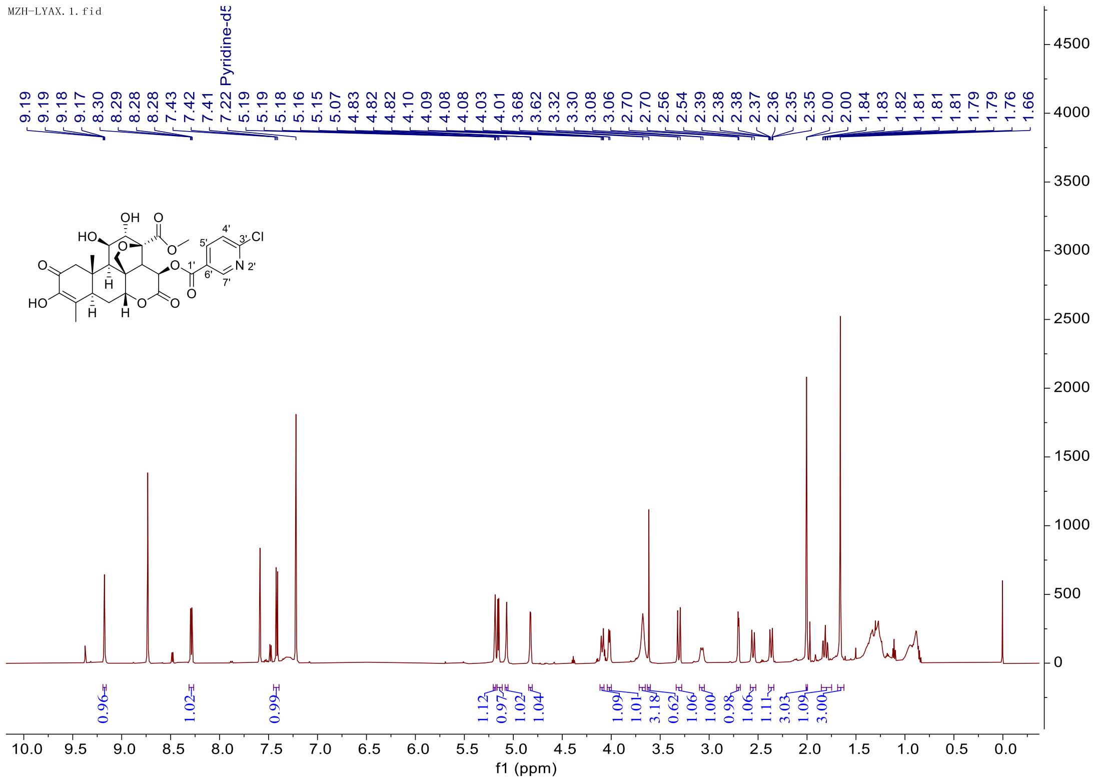

Figure S17. ^{1}H NMR spectrum of compound **6**

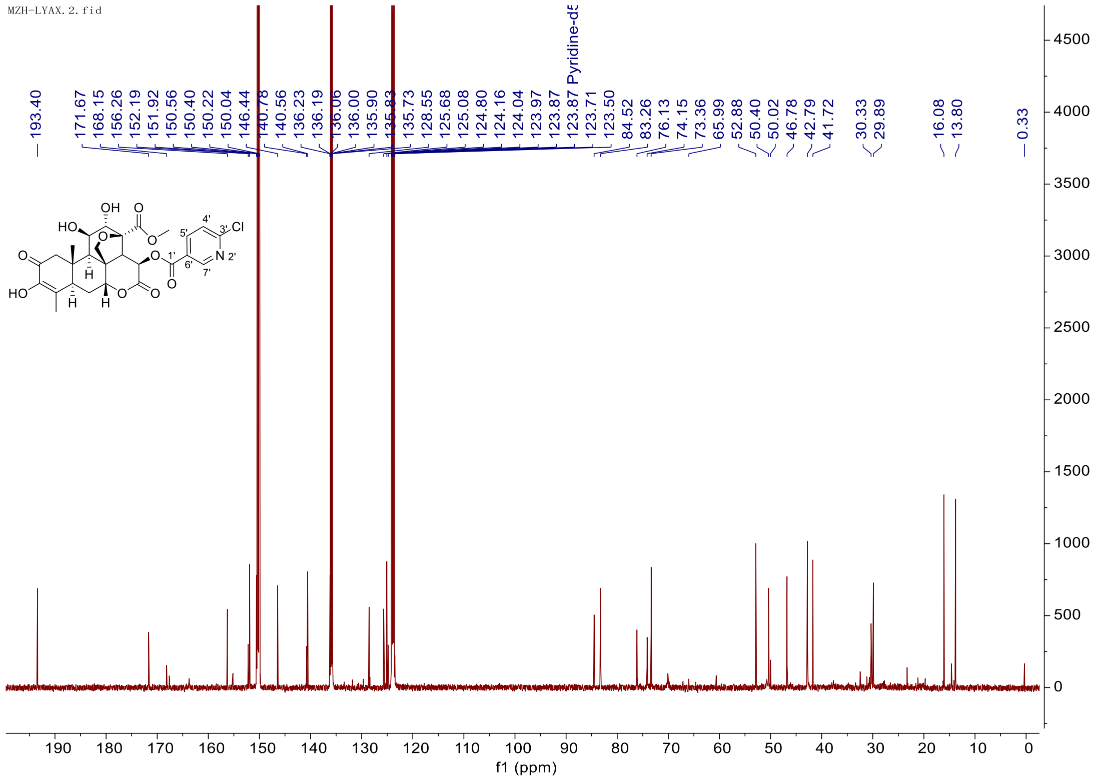

Figure S18. ^{13}C NMR spectrum of compound **6**

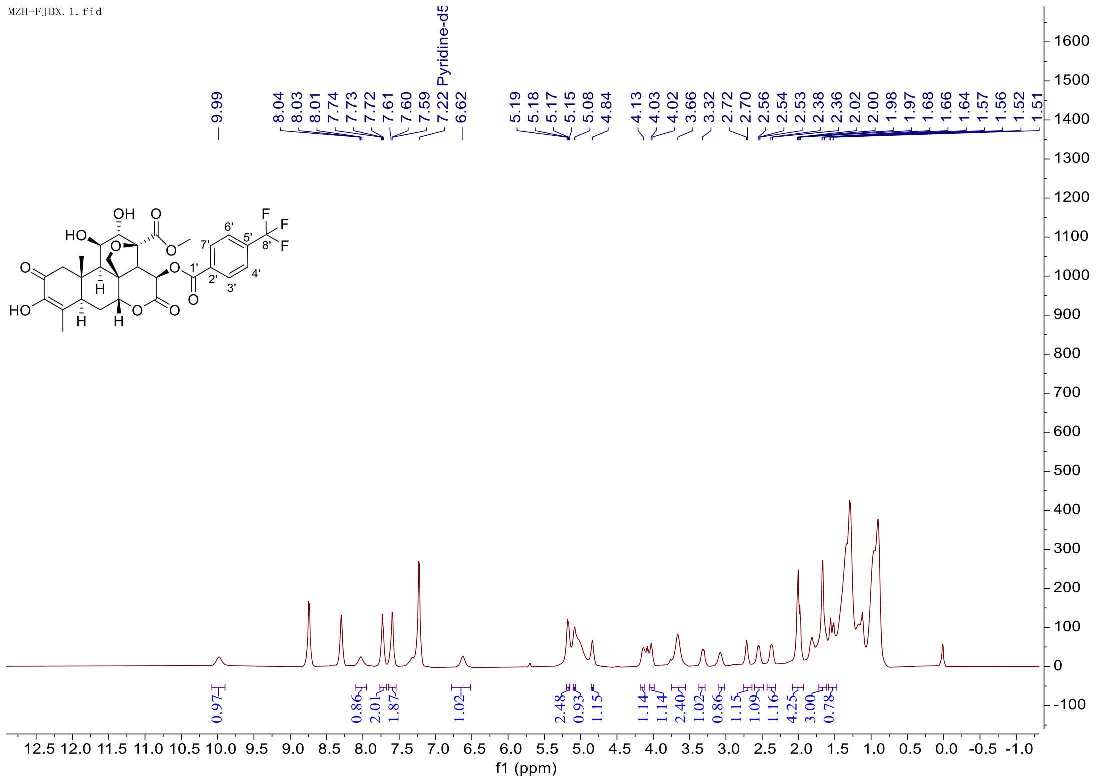

Figure S19. ^{1}H NMR spectrum of compound **7**

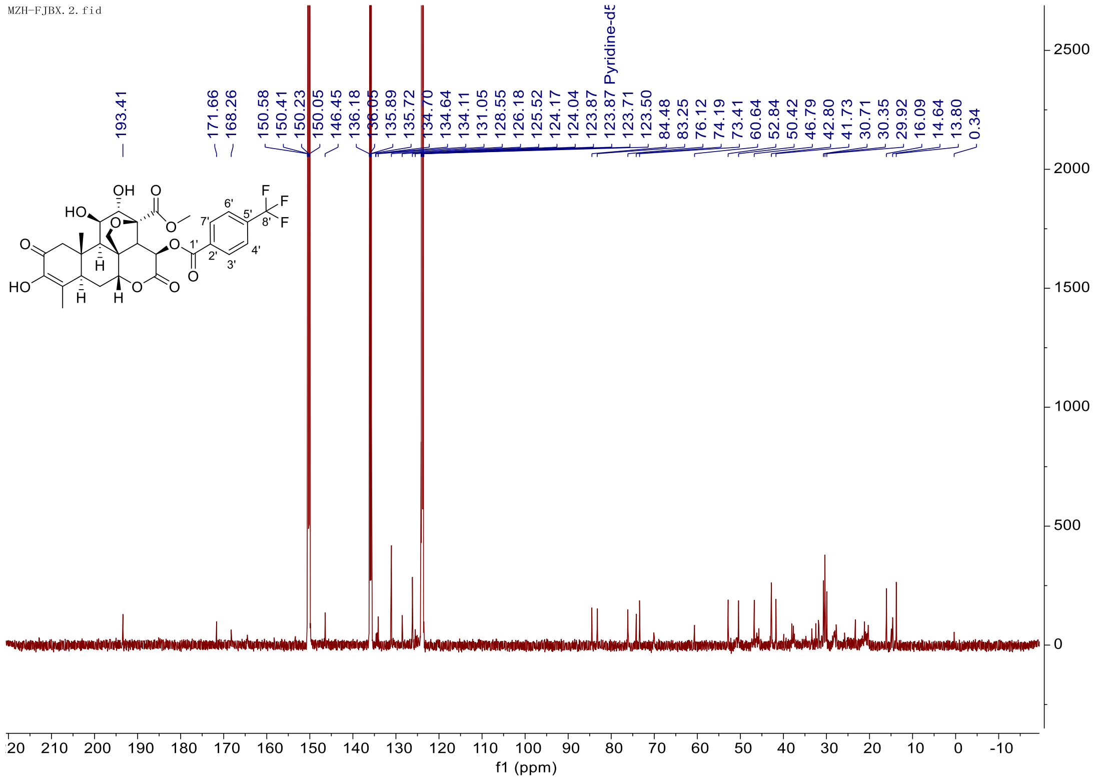

Figure S20. ^{13}C NMR spectrum of compound **7**

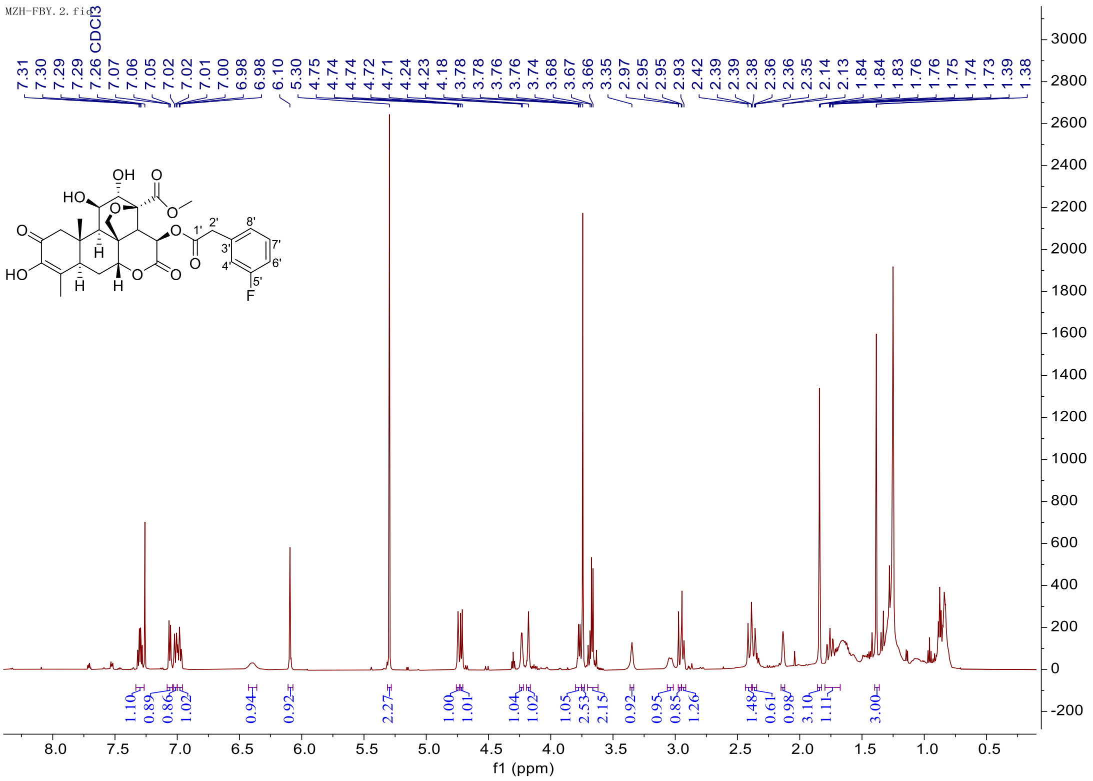

Figure S21. ^{1}H NMR spectrum of compound **8**

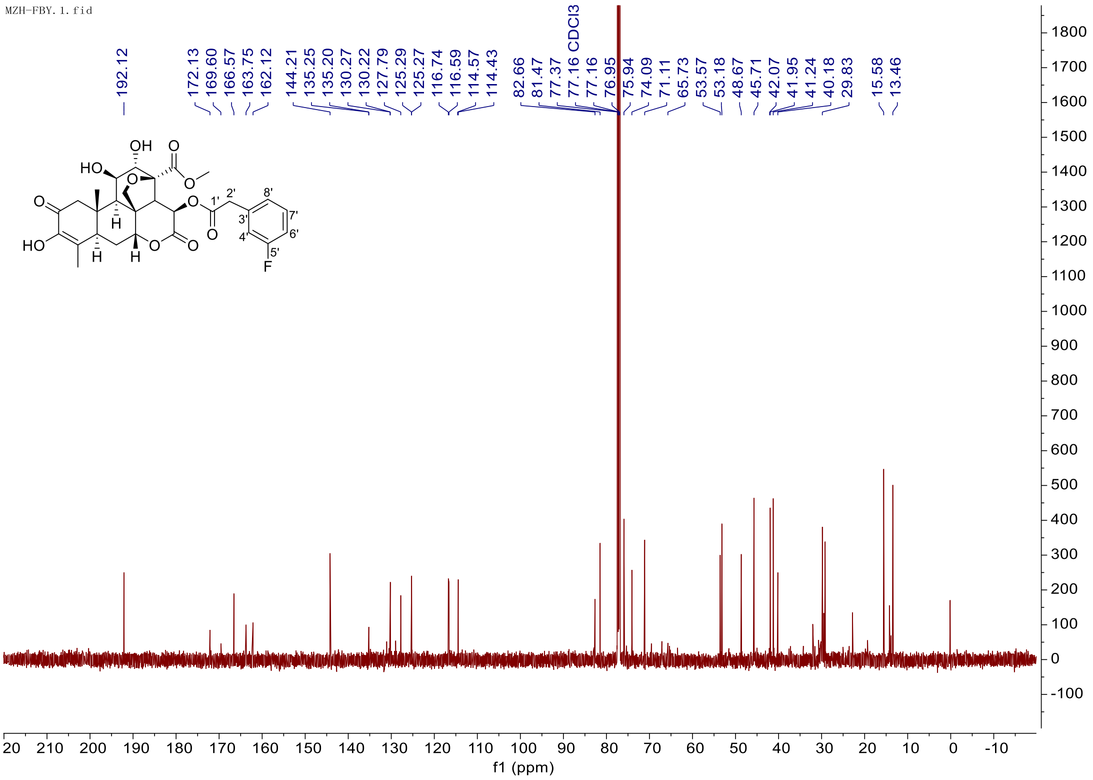

Figure S22. ^{13}C NMR spectrum of compound **8**

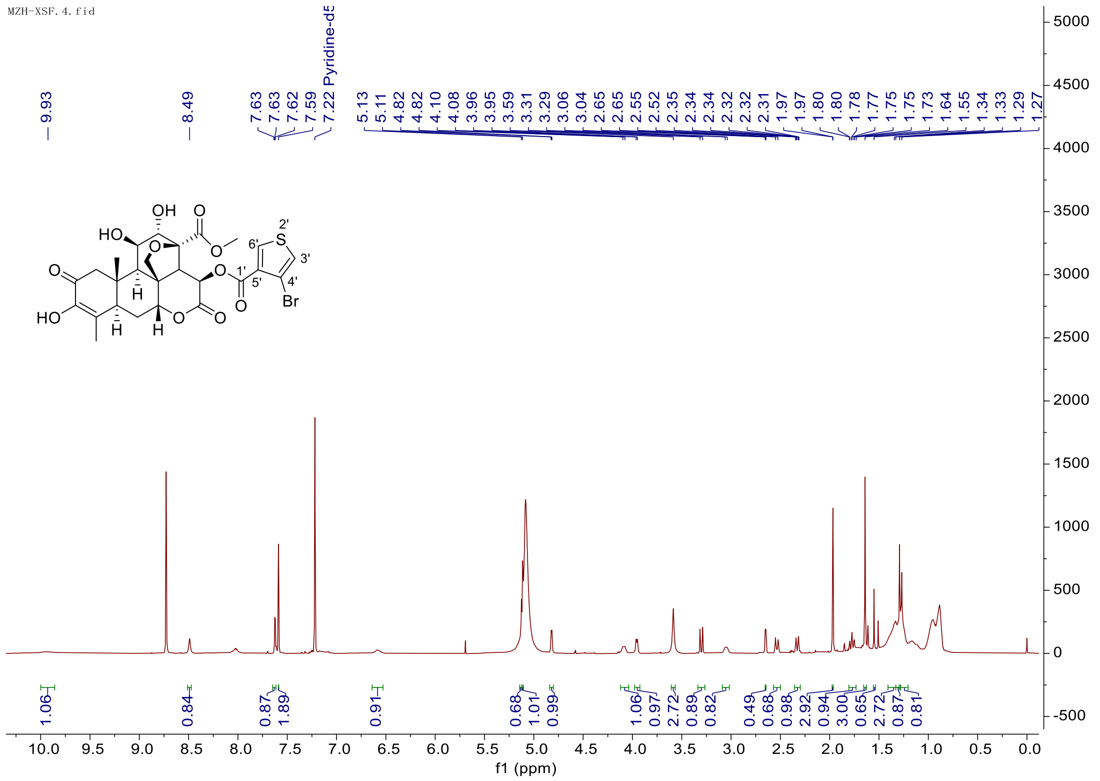

Figure S23. ^{1}H NMR spectrum of compound **9**

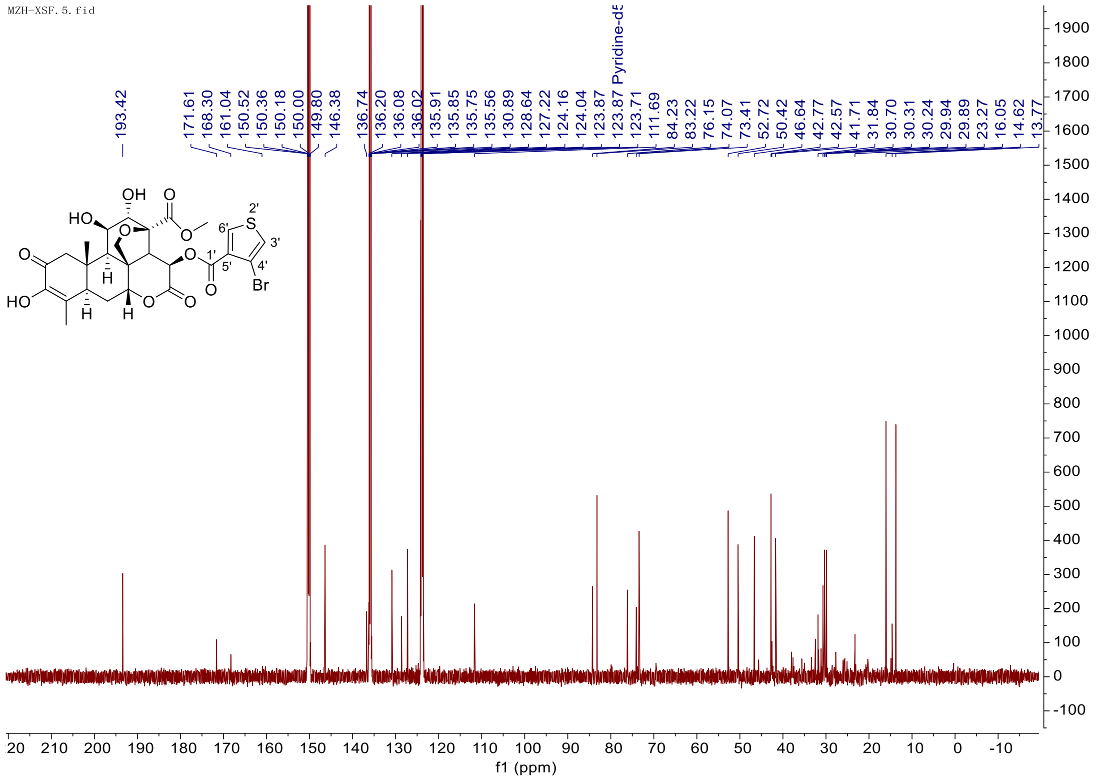

Figure S24. ^{13}C NMR spectrum of compound **9**

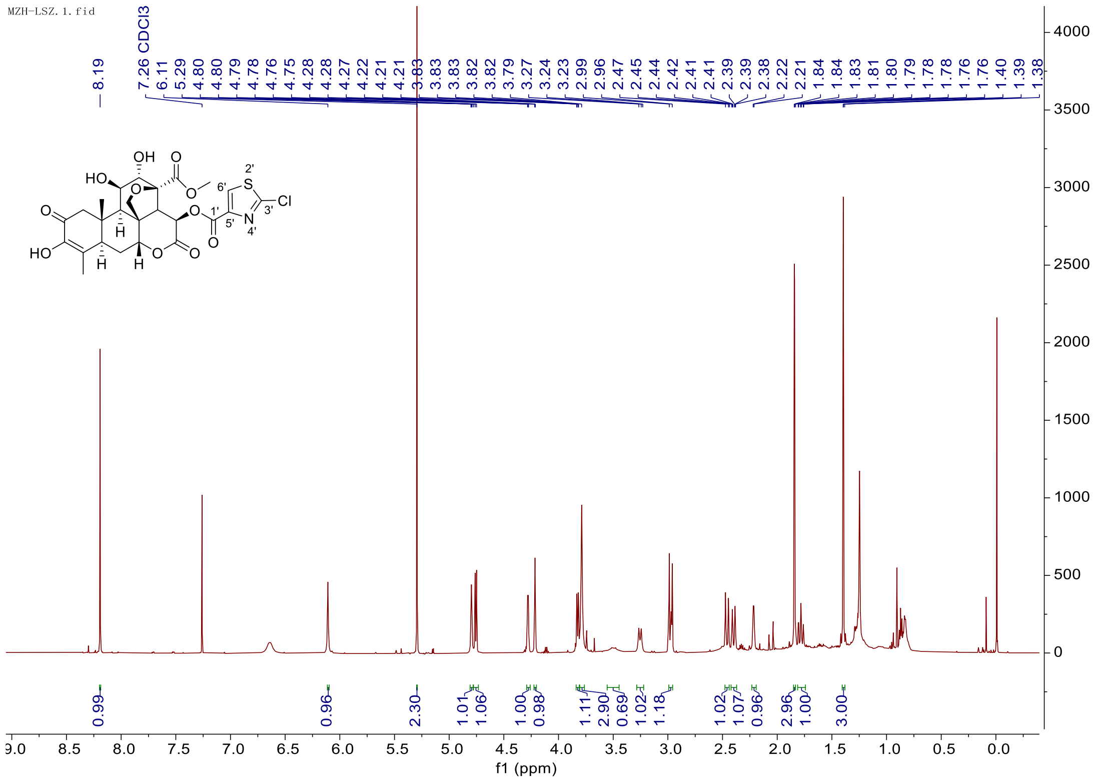

Figure S25. ^{1}H NMR spectrum of compound **10**

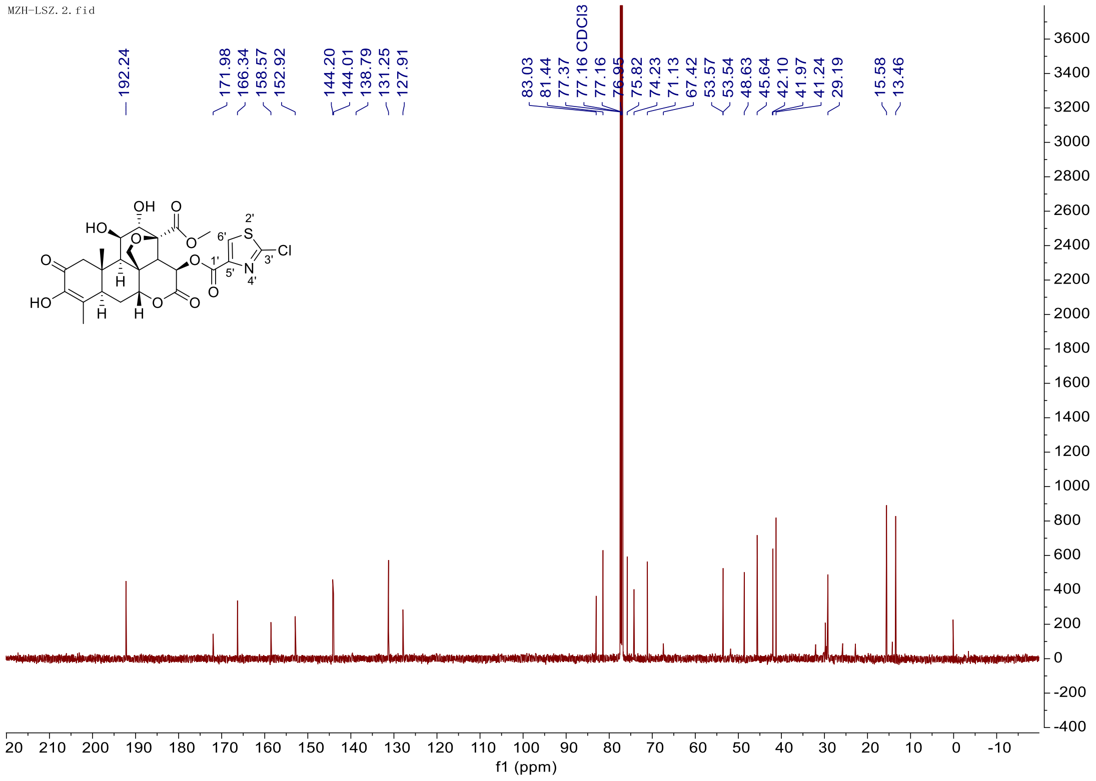

Figure S26. ^{13}C NMR spectrum of compound **10**

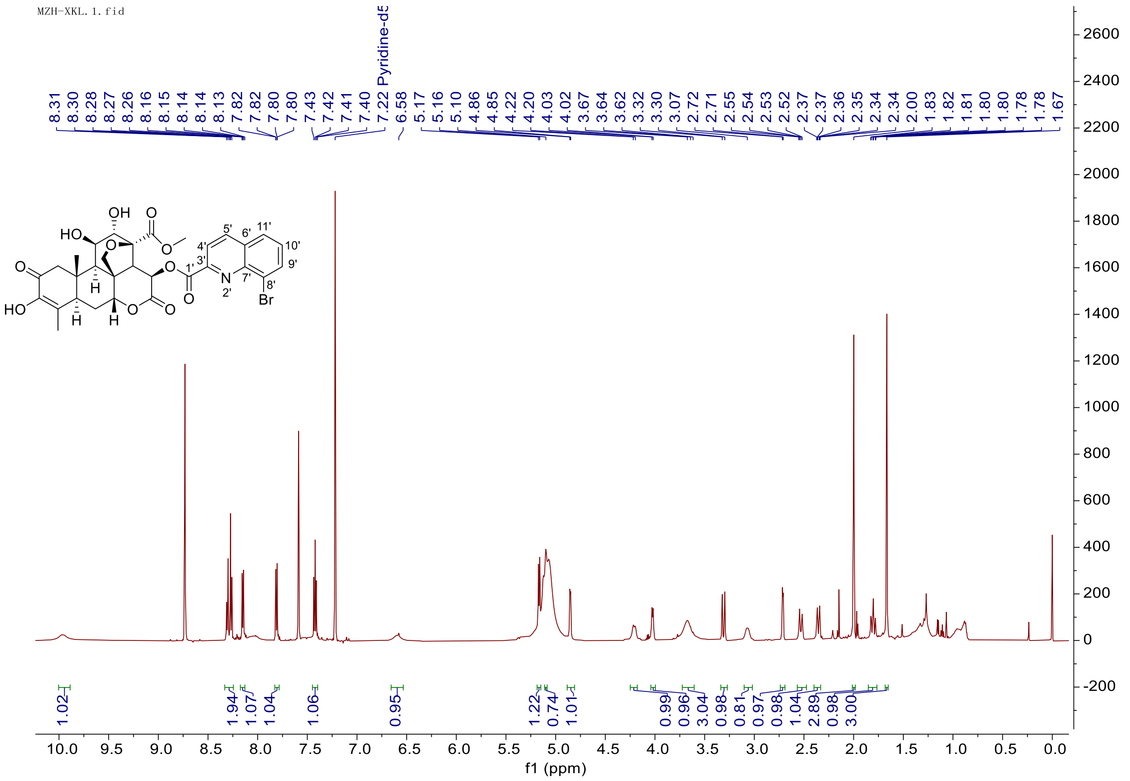

Figure S27. ^{1}H NMR spectrum of compound **11**

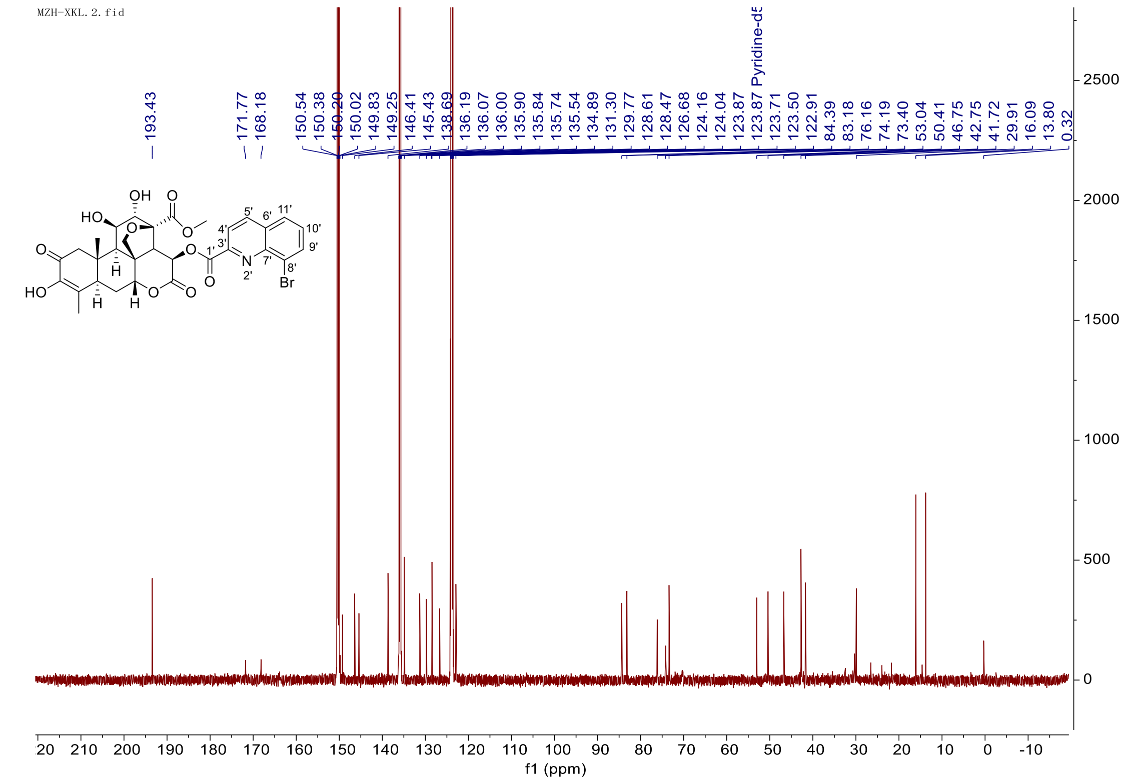

Figure S28. ^{13}C NMR spectrum of compound **11**

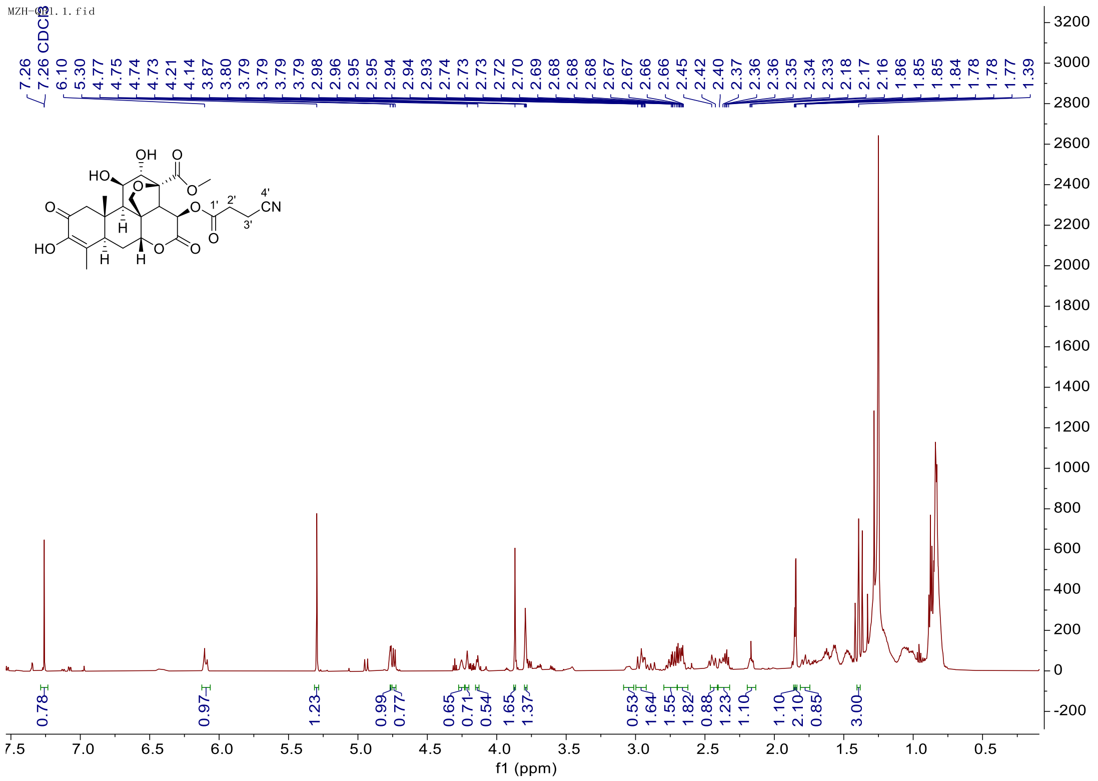

Figure S29. ^{1}H NMR spectrum of compound **12**

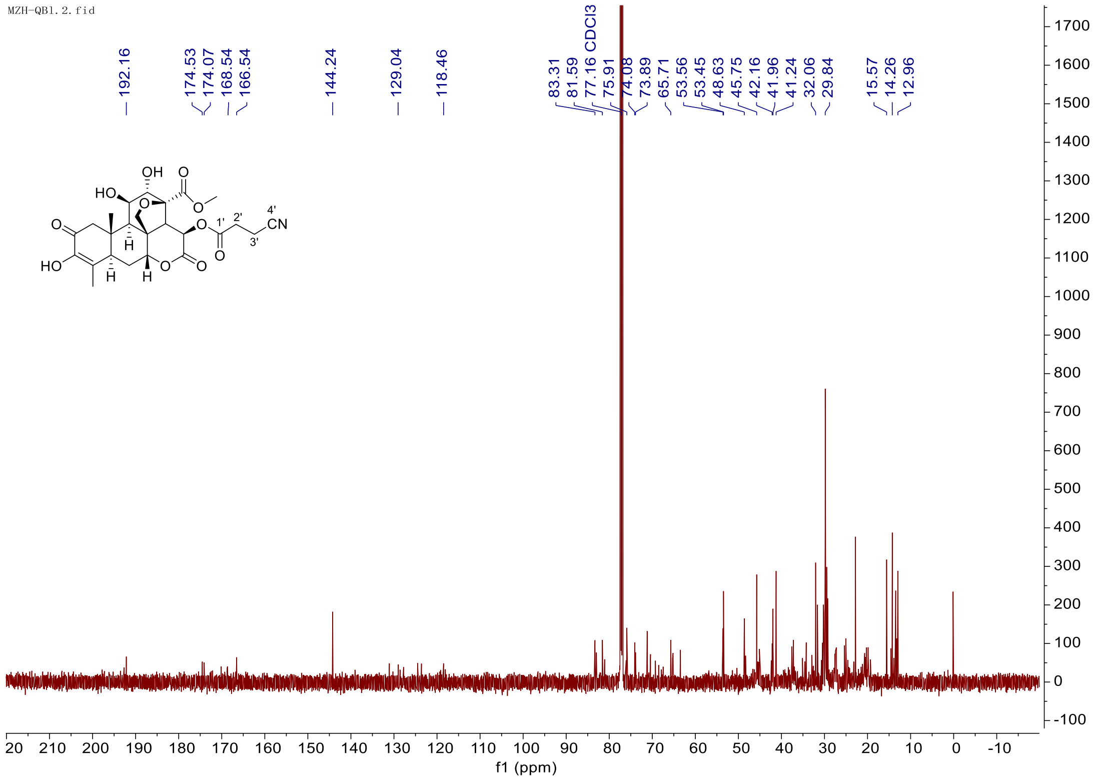

Figure S30. ^{13}C NMR spectrum of compound **12**

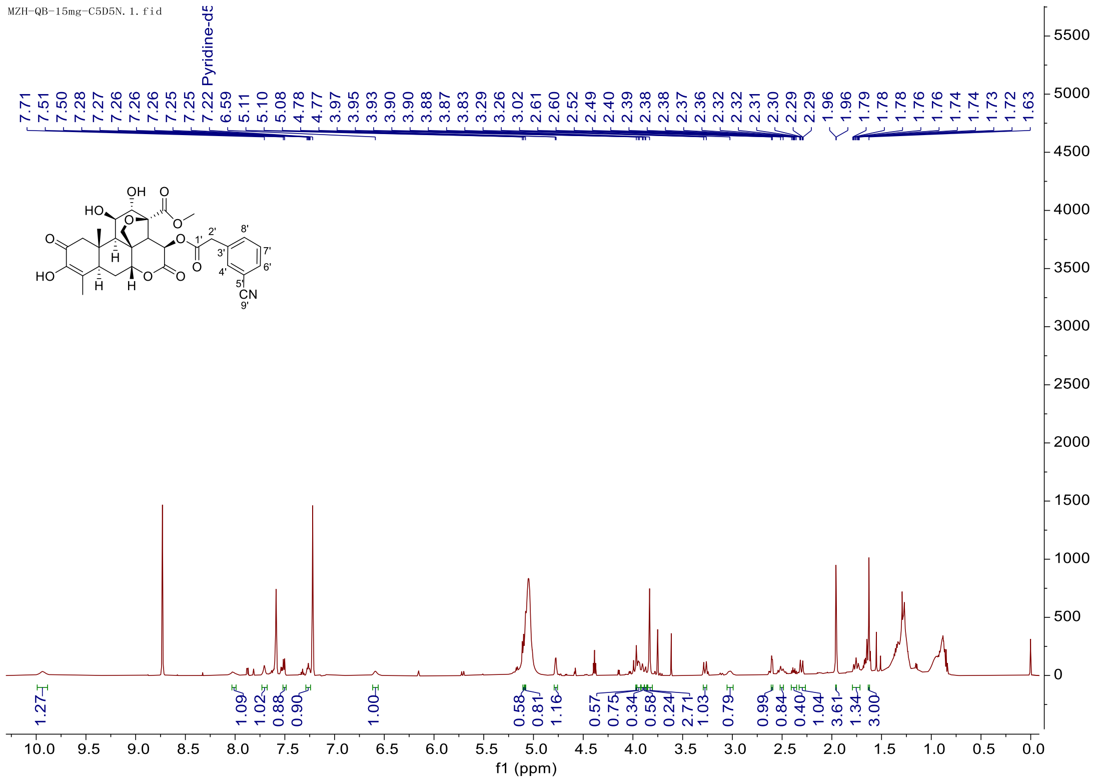

Figure S31. ^{1}H NMR spectrum of compound **12**

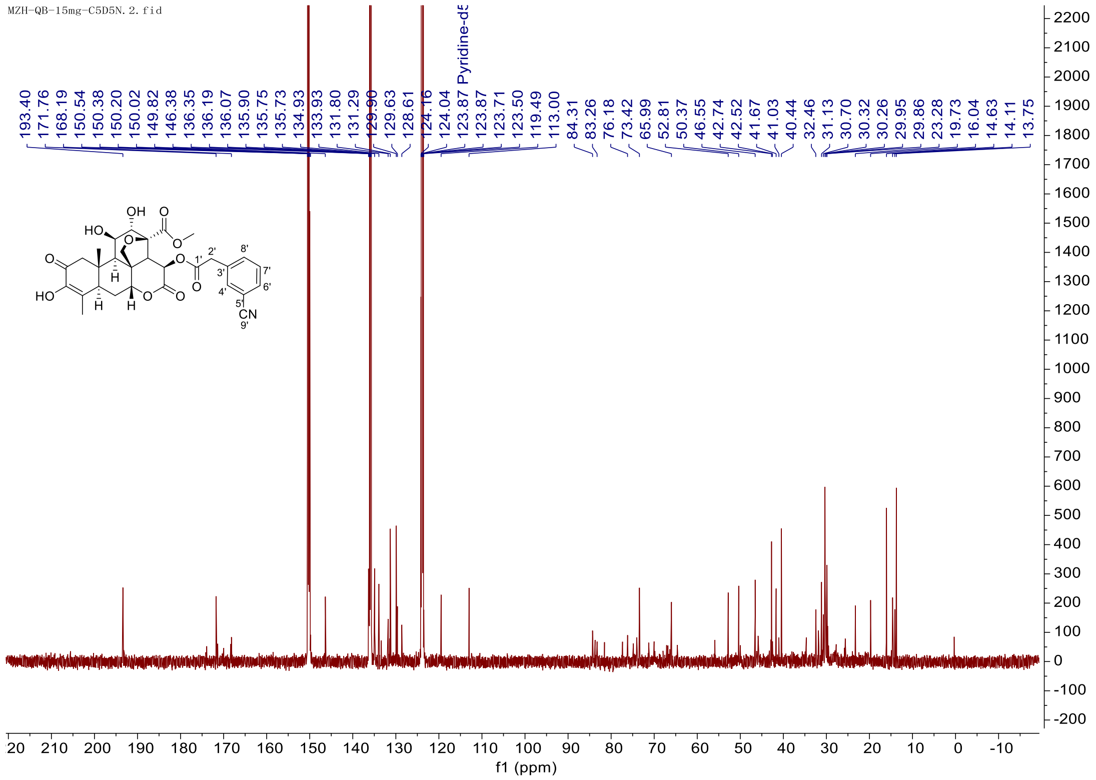

Figure S32. ^{13}C NMR spectrum of compound **12**

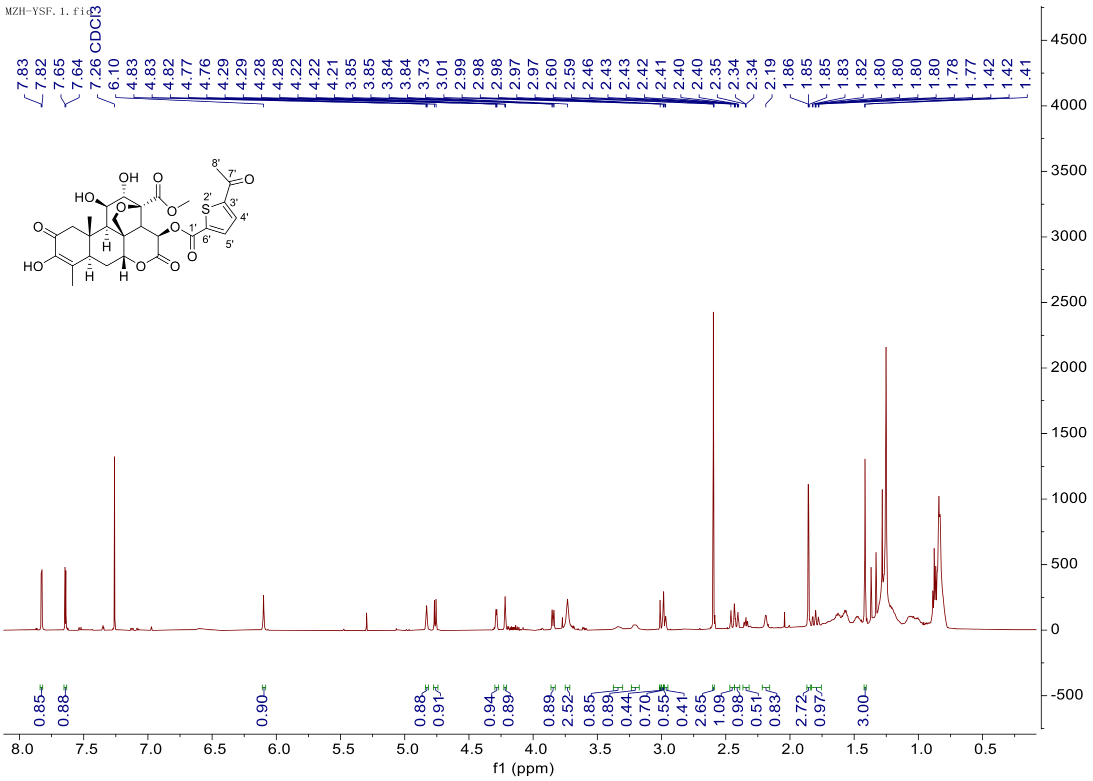

Figure S33. ^{1}H NMR spectrum of compound **14**

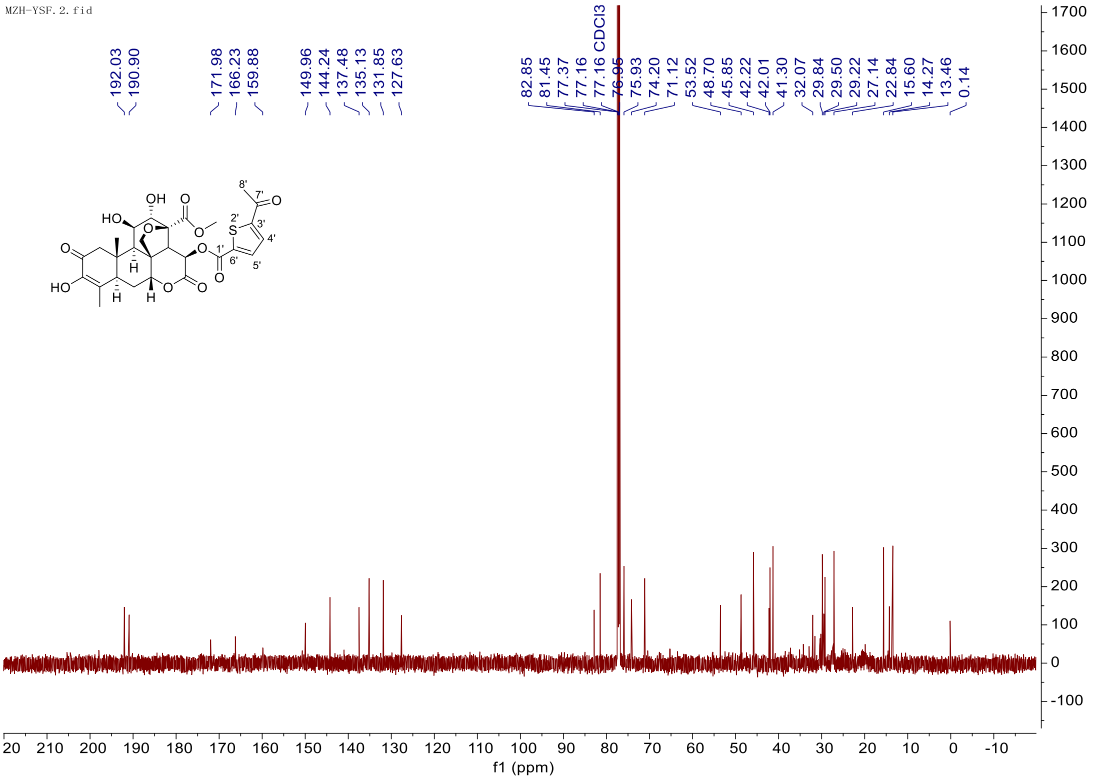

Figure S34. ^{13}C NMR spectrum of compound **14**

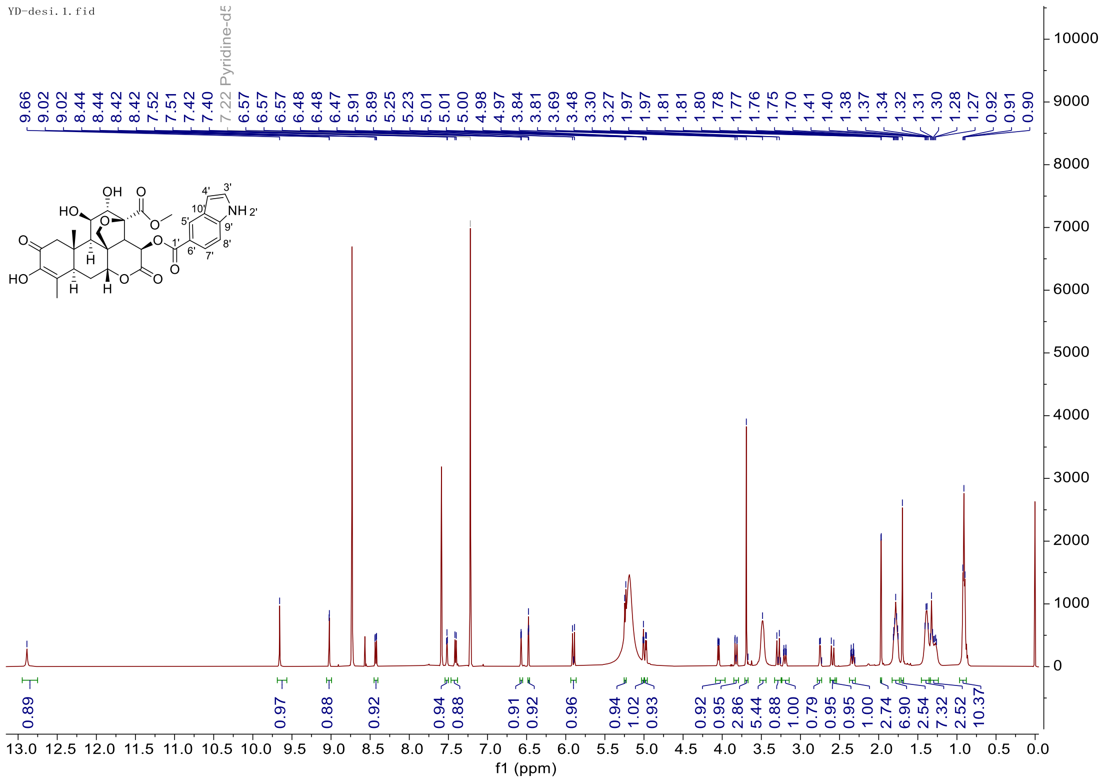

Figure S35. ^{1}H NMR spectrum of compound **15**

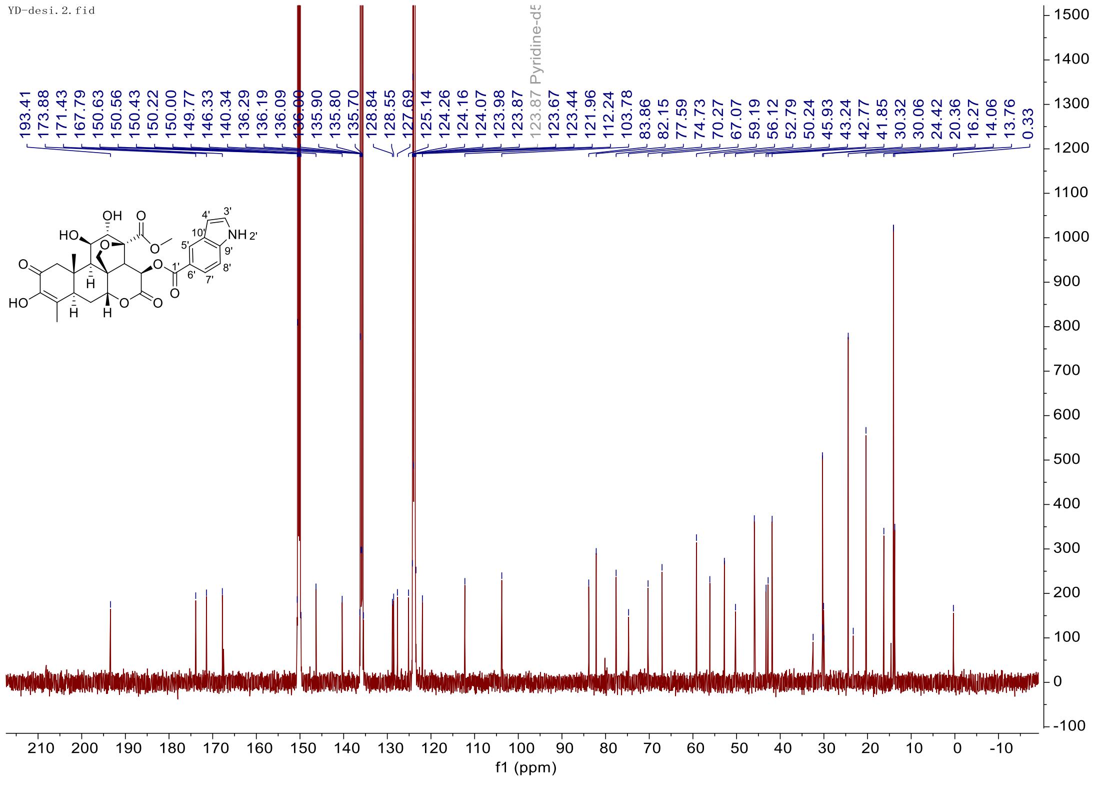

Figure S36. ^{13}C NMR spectrum of compound **15**
